# Superior antitumor immune response achieved with proton over photon immunoradiotherapy is amplified by the nanoradioenhancer NBTXR3

**DOI:** 10.1186/s12951-024-02855-0

**Published:** 2024-10-01

**Authors:** Yun Hu, Sébastien Paris, Narayan Sahoo, Qi Wang, Qianxia Wang, Hampartsoum B. Barsoumian, Ailing Huang, Jordan Da Silva, Célia Bienassis, Claudia S. Kettlun Leyton, Tiffany A. Voss, Fatemeh Masrorpour, Thomas Riad, Carola Leuschner, Nahum Puebla-Osorio, Saumil Gandhi, Quynh-Nhu Nguyen, Jing Wang, Maria Angelica Cortez, James W. Welsh

**Affiliations:** 1 Department of Radiation Oncology, The University of Texas MD Anderson Cancer Center, 6565 MD Anderson Blvd, Houston, TX 77030 USA; 2 Department of Translational Science, Nanobiotix, Paris, France; 3 Department of Radiation Physics, The University of Texas MD Anderson Cancer Center, Houston, TX USA; 4 Department of Bioinformatics and Computational Biology, The University of Texas MD Anderson Cancer Center, Houston, TX USA; 5 Department of Physics and Astronomy, Rice University, Houston, TX USA

**Keywords:** NBTXR3 nanoradioenhancer, Proton radiotherapy, Photon radiotherapy, Immunotherapy, Lung cancer

## Abstract

Recent findings suggest that immunoradiotherapy (IRT), combining photon radiotherapy (XRT) or proton radiotherapy (PRT) with immune checkpoint blockade, can enhance systemic tumor control. However, the comparative efficacy of XRT and PRT in IRT remains understudied. To address this, we compared outcomes between XRT + αPD1 and PRT + αPD1 in murine αPD1-resistant lung cancer (344SQR). We also assessed the impact of the nanoparticle radioenhancer NBTXR3 on both XRT + αPD1 and PRT + αPD1 for tumor control and examined the tumor immune microenvironment using single-cell RNA sequencing (scRNAseq). Additionally, mice cured by NBTXR3 + PRT + αPD1 were rechallenged with three lung cancer cell lines to evaluate memory antitumor immunity. PRT + αPD1 showed superior local tumor control and abscopal effects compared to XRT + αPD1. NBTXR3 + PRT + αPD1 significantly outperformed NBTXR3 + XRT + αPD1 in tumor control, promoting greater infiltration of antitumor lymphocytes into irradiated tumors. Unirradiated tumors treated with NBTXR3 + PRT + αPD1 had more NKT cells, CD4 T cells, and B cells, with fewer Tregs, than those treated with NBTXR3 + XRT + αPD1. NBTXR3 + PRT + αPD1 also stimulated higher expression of IFN-γ, GzmB, and Nkg7 in lymphocytes, reduced the TGF-β pathway, and increased tumor necrosis factor alpha expression compared to NBTXR3 + XRT + αPD1. Moreover, NBTXR3 + PRT + αPD1 resulted in greater M1 macrophage polarization in both irradiated and unirradiated tumors. Mice achieving remission through NBTXR3 + PRT + αPD1 exhibited a robust memory immune response, effectively inhibiting growth of subsequent tumors from three distinct lung cancer cell lines. Proton IRT combined with NBTXR3 offers enhanced tumor control and survival rates over photon-based treatments in managing αPD1-resistant lung cancer, indicating its potential as a potent systemic therapy.

**Graphical Abstract:**

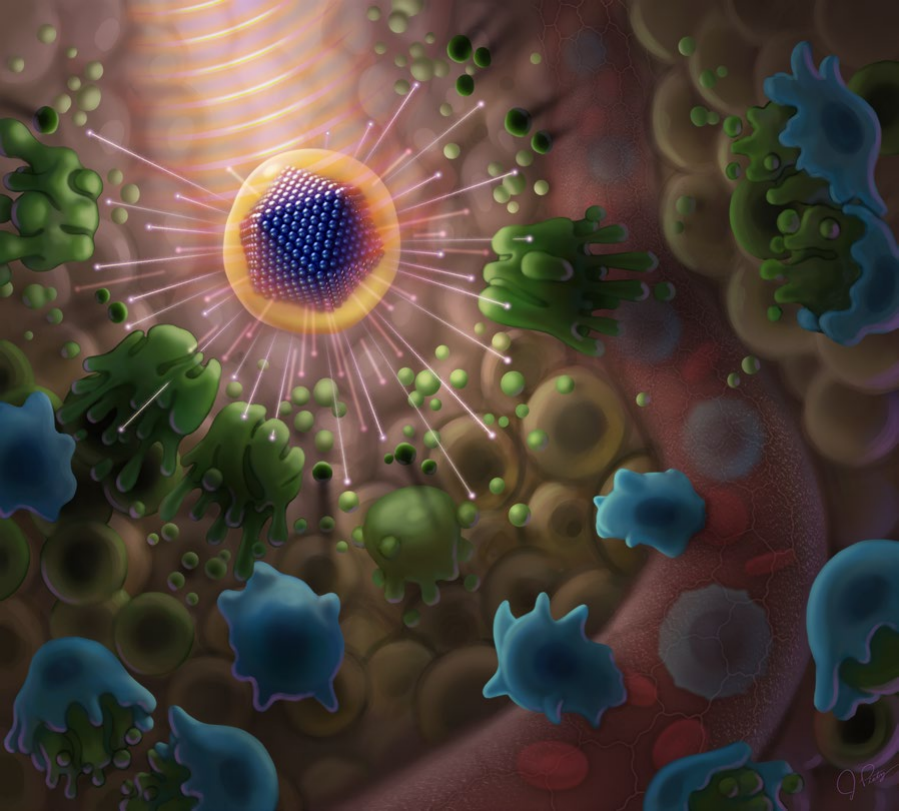

**Supplementary Information:**

The online version contains supplementary material available at 10.1186/s12951-024-02855-0.

## Introduction

Currently, photon radiotherapy (XRT) is predominantly utilized in clinical practice owing to its cost-effectiveness and extensive accessibility. On the other hand, despite its associated higher costs and restricted availability primarily in developed regions, proton radiotherapy (PRT) is known for its association with fewer side effects [1, 2]. Intriguingly, a multitude of clinical trials suggests a parity in treatment efficacy between XRT and PRT [3, 4]. Recent investigations, including our own, have shed light on the expanding role of immunoradiotherapy (IRT)—an amalgamation of radiotherapy and immune checkpoint blockade [5–11]. This advancement has propelled the utility of radiotherapy beyond the confines of local tumor control to a more holistic systemic disease management approach.

Ionizing radiation is known to induce immunogenic cell death in tumor cells, which subsequently mobilizes antitumor immune cells towards the irradiated site [12]. This crucial process paves the way for these immune cells to disseminate to distant tumors, thereby playing a significant role in controlling metastasis [13]. The distinctive Bragg peak characteristic of PRT permits more precise deposition of radiation energy into tumors compared to the exit radiation dosage of XRT [14]. This precision potentially provides a more advantageous platform for instigating antitumor immune responses. By diminishing collateral damage to the surrounding lymph nodes and curtailing radiation-induced lymphopenia, PRT might contribute significantly to the preservation of immune cells within the bloodstream [15].

Despite these advantages, the existing scientific literature is notably deficient in studies that compare the efficacy of proton IRT and photon IRT. Given the increasing clinical application of IRT and the global growth of proton therapy centers, it is crucial to comprehend the therapeutic distinctions and the fundamental mechanisms that differentiate proton IRT from photon IRT. In order to bridge this knowledge gap, we conducted a comparative study of the treatment outcomes of proton IRT and photon IRT using a mouse model of anti-PD1 (αPD1)-resistant lung cancer [16]. The prevalence of αPD1 resistance among cancer patients poses a significant challenge to the successful application of immune checkpoint blockade [16, 17]. Our prior research suggested that a dual-therapy approach employing photon IRT may fall short in effectively controlling systemic tumors exhibiting resistance to αPD1 [8]. The incorporation of NBTXR3 into photon IRT triggered an abscopal effect in αPD1-resistant tumors in mice, leading to augmented treatment outcomes in cancer patients [8, 18–20]. NBTXR3 is composed of hafnium oxide nanoparticles designed to enhance radiation, delivered to patients by intratumoral injection, has attained approval for the treatment of sarcoma in Europe and is currently under investigation in worldwide clinical trials as part of a combination regimen with IRT [21]. Consequently, in this current study, we broadened our comparative analysis to encompass the therapeutic outcomes between the amalgamation of NBTXR3 and photon IRT, and NBTXR3 and proton IRT. Previous research indicated that both NBTXR3 + photon IRT and NBTXR3 + proton IRT could improve antitumor immune cell infiltration and stimulate antitumor immune pathways in both irradiated and unirradiated tumors [8, 18, 19, 22, 23]. To mechanistically dissect the differential impacts of NBTXR3 + photon IRT and NBTXR3 + proton IRT on the modulation of the tumor immune microenvironment (TIME), we employed single-cell RNA sequencing (scRNAseq) for tumor analysis. Additionally, we assessed the capability of NBTXR3 + proton IRT-treated mice to resist tumor relapse by challenging them with three distinct lung tumor cell lines. This research yielded several significant observations: (1) Proton IRT demonstrated significantly superior local and distant tumor control than photon IRT; (2) The addition of NBTXR3 to either proton IRT or photon IRT improved treatment outcomes; (3) NBTXR3 + proton IRT resulted in significantly better tumor control and survival than NBTXR3 + photon IRT; (4) NBTXR3 + proton IRT induced a more potent antitumor immune response than NBTXR3 + photon IRT in both irradiated and unirradiated tumors; (5) Mice cured by NBTXR3 + proton IRT maintained a potent antitumor memory immune response and effectively rejected rechallenge by different lung cancer cells. These findings could provide valuable guidance for clinical applications and further insights into the mechanistic differences between proton IRT and photon IRT and emphasize the benefit of using NBTXR3 to improve both modalities.

## Materials and methods

### Materials

NBTXR3 nanoparticles were generously supplied by Nanobiotix, and αPD1 was provided by Bristol Myers Squibb. We procured flow cytometry antibodies from BioLegend, including αCD45–Pacific Blue (catalog 103126), αCD4–APC/Fire 750 (catalog 100568), αCD8–PerCP-Cy5.5 (catalog 100734), αCD62L–PE-Cy7 (catalog 104418), and αCD44-APC (catalog 103012). The Bouin’s fixative solution used for staining lung metastases was obtained from Polysciences Inc. (catalog 16045-1).

### Cell line and culture

The αPD1-resistant mouse lung cancer cell line 344SQR, developed in a previous study, was utilized in this investigation to assess the efficacy of various forms of IRT [16]. In our tumor rechallenge study, in addition to 344SQR, we utilized two specific mouse lung cancer cell lines: 344SQP, which is an αPD1-sensitive cell line and the progenitor of the 344SQR line, and 393P, a non-metastatic lung cancer cell line [24]. The cultivation conditions for these three cell lines were identical to those delineated in our previous studies [8, 18]. In brief, the cell lines were cultured in complete medium composed of RPMI 1640 supplemented with 100 units/mL penicillin, 100 μg/mL streptomycin, and 10% heat-inactivated fetal bovine serum. Cultures were incubated at 37  C in an atmosphere of 5% CO_2_.

### Tumor establishment and IRT treatment

All the mice used in this study were female 129/SvEv mice, aged 8–12 weeks, homozygous for non-wild-type alleles, and procured from Taconic Biosciences. The bilateral tumor model was established via methods outlined in previous studies [8, 18, 19]. In brief, the mice were injected with 5 × 10^4^ 344SQR cells into the right leg on day 0 to form primary tumors (which would undergo irradiation) and the same number of 344SQR cells were administered into the left legs on day 4 to establish secondary tumors. The mice (n = 7–10) were randomly allocated to the following treatment groups: 1. Control, 2. XRT + αPD1, 3. NBTXR3 + XRT + αPD1, 4. PRT + αPD1, and 5. NBTXR3 + PRT + αPD1. The control group mice did not receive radiotherapy, αPD1, or NBTXR3. On day 7, the primary tumors in the groups receiving NBTXR3 were intratumorally (I.T.) injected with NBTXR3 nanoparticles, constituting 25% of the tumor volume. This was followed by two fractions of 12 Gy radiation (total dose of 24 Gy) on days 8 and 9, employing a 200 MeV proton beam from a Hitachi PROBEAT (Hitachi America, Ltd.) at the MD Anderson Proton Therapy Center, or a PXi X-Rad SmART irradiator. Photon radiation was administered using two opposing beams, aligned in anteroposterior and posteroanterior positions, and employed a 15-mm circular collimator for precise targeting. The collimators were initially commissioned by Precision XRay Corporation during installation. To maintain accuracy and consistency in treatment plans, routine output verifications were conducted using an ion chamber. These checks ensured that the radiation outputs remained stable and unchanged over time [8]. Proton beam irradiation of tumors in mice legs was carried out by placing them at the center of the spread-out Bragg peak (SOBP) of a passively scattered 200 MeV proton beam. This beam has distal 90% range of 19 cm, and a SOBP width of 10 cm was chosen for this irradiation with tumor placed at the depth of 14 cm. The dose averaged linear energy transfer (LET) in this region was estimated to be around 2.0 keV/μm. A fixed radiobiological effectiveness (RBE) of 1.1 for protons was used to covert the proton dose to its equivalent photon dose as per the current clinical practice in proton therapy. A half-beam blocked field of size 18 cm × 9 cm was used to irradiate the tumors in the animal leg. The animal body was placed in the blocked part of the treatment field. A fixed source to tumor distance of 270 cm was used in the irradiation. The dose/MU was determined using a calibrated parallel plate ionization chamber (PTW Markus) placed at the 14 cm depth in the same geometry as used in animal irradiation, which was then used for determining the MU for the animal irradiation. Mice were administered two hundred micrograms of αPD1 via intraperitoneal injections on days 7, 10, 14, 21, 28, 35, and 42. The tumors were consistently monitored, and their volumes were calculated as V = 0.5 × width^2^ × length. All animal procedures followed protocols approved by the Institutional Animal Care and Use Committee at MD Anderson Cancer Center.

### Tumor harvest and scRNAseq

Primary tumors from the Control, NBTXR3 + XRT + αPD1, and NBTXR3 + PRT + αPD1 groups (n = 5), along with secondary tumors (n = 5) from the Control, XRT + αPD1, NBTXR3 + XRT + αPD1, PRT + αPD1, and NBTXR3 + PRT + αPD1 groups, were collected on day 17. Tumor tissues were cut into small pieces and digested with 250 µg/mL of Liberase (Roche, cat. #05401127001) and 20 µg/mL DNAse (Sigma-Aldrich, cat. #4716728001) at 37 °C for 30 min. The digestion process was stopped with 1 mL fetal bovine serum and the samples were filtered. The dissociated cells from each mouse in the same group were pooled and stained with αCD45-FITC, then washed with RPMI 1640 medium supplemented with 2% fetal bovine serum (FBS), followed by sorting with a BD FACSAria II cell sorter. After flow sorting, at least 1 × 10^5^ CD45 + cells with at least 85% viability were used for scRNAseq. scRNAseq sample processing adhered to the 10 × Genomics’ 5′ scRNAseq and TCR enrichment protocols. Quality was assessed using a Qubit HS dsDNA Assay and Agilent HS DNA Bioanalyzer, with library concentrations confirmed via qPCR. Libraries were normalized to 5 nM and pooled at a 5:1 gene-to-TCR ratio, then sequenced on an Illumina NovaSeq 6000 using specified cycle settings. Data were analyzed with the R Seurat package, filtering out cells based on mitochondrial content and Ptprc expression, followed by data integration and PCA. Cell clusters were identified using Seurat tools and visualized via UMAP, employing ImmGenData for cell identification, focusing on CD45 + cells, and excluding nonimmune types. Marker genes were used for cell type verification and to identify differentially expressed genes [23].

### Lung metastases counting

Lungs were harvested on day 17 and preserved in Bouin’s fixative solution (Polysciences, Warrington, PA; Cat. #16,045-1) for three days. Subsequently, lung metastatic nodules were counted [8, 18, 19].

### Memory immune cell profiling

Mouse blood was collected 93 days post-PRT and circulating immune cells were stained using αCD45–Pacific Blue, αCD3-BV510, αCD4–APC/Fire 750, αCD8–PerCP-Cy5.5, αCD62L–PE-Cy7, and αCD44-APC. The stained samples were analyzed using a Gallios Flow Cytometer (Beckman Coulter), and the flow cytometry data were processed using Kaluza software version 2.1. [23].

### Tumor rechallenge

On day 102 following PRT, the four surviving mice in the NBTXR3 + PRT + αPD1 group underwent a rechallenge with 5 × 10^4^ 344SQR cells, administered on the right flank. Subsequently, on day 157 post-PRT, these four mice were subjected to an additional challenge involving 5 × 10^5^ 344SQP cells, introduced to the left flank. Then, 247 days after PRT, the survivor mice were challenged with 5 × 10^5^ 393P cells on the right flank.

To serve as a control group, five untreated mice were separately injected with 344SQR, 344SQP, and 393P cells. Tumor volumes were calculated using the formula V = 0.5 × width^2^ × length. When a tumor reached a size of 14mm, the mice were humanely euthanized.

### Statistical analysis

The statistical analysis was performed using previously described methods [23]. Briefly, tumor volumes are presented as mean tumor volume ± standard error of the mean (SEM) and were evaluated using two-way analysis of variance (ANOVA). Survival rates of mice were analyzed employing the Kaplan–Meier method and compared through log-rank tests. The number of lung metastases and flow immune cell populations were examined using two-tailed t tests. The scRNAseq data were compared using either ordinary one-way ANOVA or the Kruskal–Wallis test. All data are expressed as mean ± SEM. A P-value less than 0.05 was deemed statistically significant.

## Results

### Local and systemic tumor control achieved photon IRT is outperformed by proton IRT and amplified by NBTXR3

**Fig. 1:**
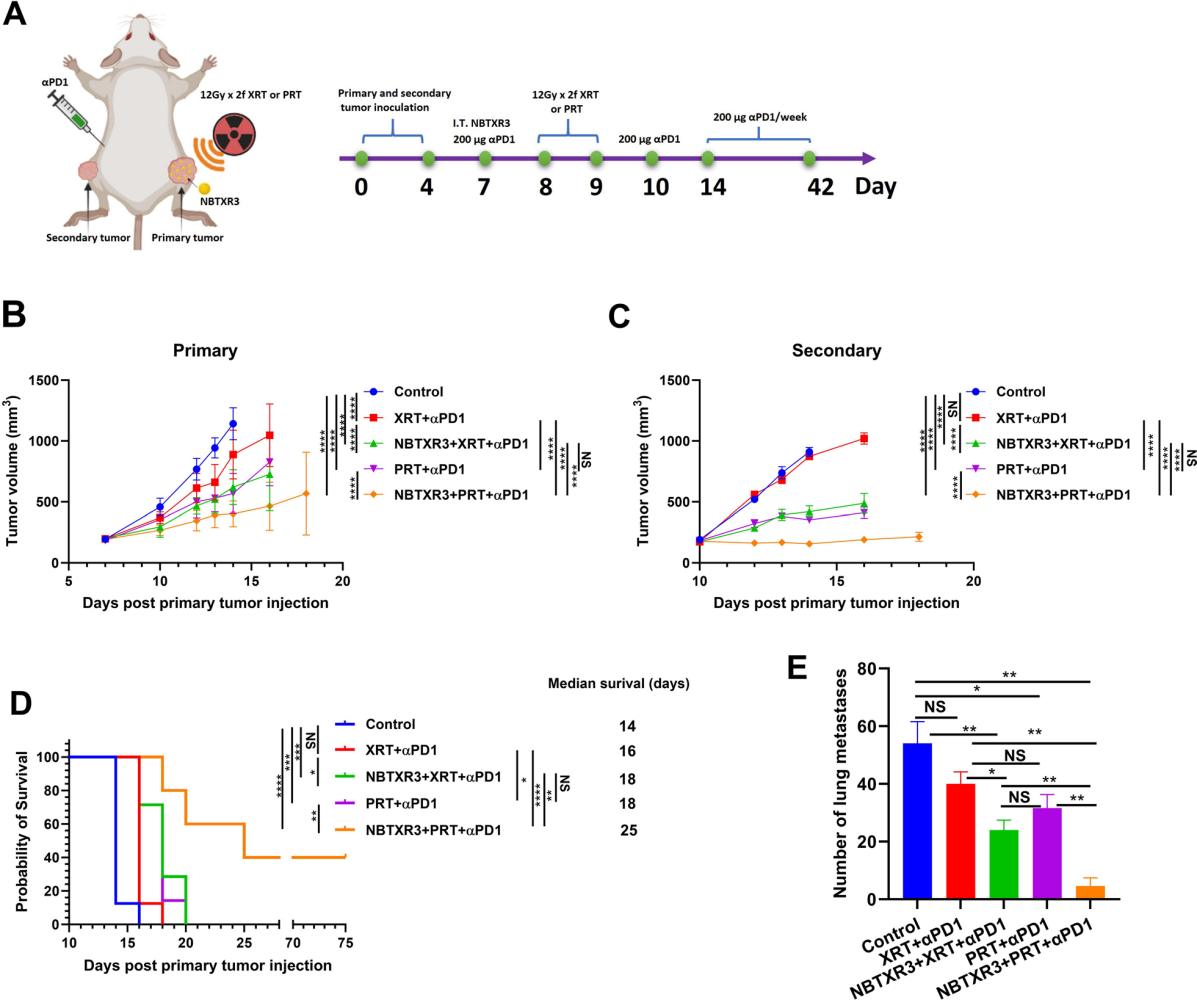
Superior tumor control achieved by proton IRT over photon IRT. **A** Depiction of treatment scheme combining NBTXR3, XRT, PRT, and αPD1 in a murine model of αPD1-resistant lung cancer. **B** Mean growth trajectories of irradiated tumors (n = 7–10). **C** Mean growth trajectories of unirradiated tumors (n = 8–10). **D** Survival rates and median survival duration. **E** Count of lung metastases (N = 5). Female 129 Sv/Ev mice aged 8–12 weeks were subcutaneously injected with 344SQR αPD1-resistant lung cancer cells on the right leg (day 0) and the left leg (day 4) to establish primary and secondary tumors, respectively. NBTXR3 radio-enhancing nanoparticles were administered intratumorally into the primary tumors on day 7, followed by two 12Gy doses of photon or proton radiation. Intraperitoneal injection of 200 μg αPD1 was performed on days 7, 10, 14, 21, 28, 35, and 42. Tumor volumes were compared using 2-way ANOVA, and mouse survival rates were assessed using the Kaplan–Meier method, with differences compared via log-rank tests. The count of lung metastases was compared using 2-tailed t-tests. Data are displayed as mean ± SEM. Statistical significance was set at P < 0.05. *P < 0.05; **P < 0.01; ***P < 0.001; ****P < 0.0001. NS, not significant

To evaluate the therapeutic efficacy of PRT + αPD1 versus XRT + αPD1, and NBTXR3 + XRT + αPD1 versus NBTXR3 + PRT + αPD1, a dual-tumor mouse model was employed using 344SQR αPD1-resistant lung cancer cell line. As illustrated in Fig. 1A, the primary tumor was either treated or untreated with an intratumoral injection of NBTXR3 and subsequently irradiated with two fractions of 12 Gy (accumulating a total dosage of 24 Gy) by XRT or PRT. The secondary tumor, conversely, received neither irradiation nor NBTXR3 injection. Thereafter, mice were subjected to multiple rounds of αPD1 treatment.

Consistent with preceding observations [8, 23], either XRT or PRT in conjunction with αPD1 markedly postponed growth in irradiated tumors (Fig. 1B and Supplemental Fig. 1). Intriguingly, PRT + αPD1 demonstrated superior control over irradiated tumor growth compared to XRT + αPD1. Furthermore, the incorporation of NBTXR3 with XRT + αPD1 and PRT + αPD1 significantly enhanced the control of irradiated tumor growth, affirming our earlier observations [8, 23].

Within unirradiated tumors, PRT + αPD1 was observed to significantly retard tumor growth compared to XRT + αPD1 (Fig. 1C and Supplemental Fig. 1). It is important to note that while XRT + αPD1 did not successfully induce an abscopal effect, PRT + αPD1 achieved this outcome. Incorporating NBTXR3 with XRT + αPD1 and PRT + αPD1 also significantly improved secondary tumor control. Notably, NBTXR3 + XRT + αPD1 achieved a treatment efficacy comparable to PRT + αPD1 for both irradiated and unirradiated tumors (Fig. 1B and 1C). However, NBTXR3 + PRT + αPD1 outperformed NBTXR3 + XRT + αPD1 in controlling growth in both tumors (Fig. 1B and 1C).

The superior tumor control demonstrated by PRT + αPD1 and NBTXR3 + PRT + αPD1 in comparison to XRT + αPD1 and NBTXR3 + XRT + αPD1, subsequently resulted in a significant prolongation of survival. The median survival times were recorded as 14, 16, 18, 18, and 25 days for Control, XRT + αPD1, NBTXR3 + XRT + αPD1, PRT + αPD1, and NBTXR3 + PRT + αPD1, respectively. Most notably, a 40% survival rate was achieved with NBTXR3 + PRT + αPD1, while all other treatments were unable to cure any of the mice (Fig. 1D).

Furthermore, the number of lung metastases was also counted on day 17. As depicted in Fig. 1E, lung metastases counts were 54 ± 8, 40 ± 5, 24 ± 4, 32 ± 5, 5 ± 3 for Control, XRT + αPD1, NBTXR3 + XRT + αPD1, PRT + αPD1, and NBTXR3 + PRT + αPD1 groups, respectively. All therapies, except for XRT + αPD1, significantly reduced the number of lung metastases compared to the Control group. Furthermore, NBTXR3 + XRT + αPD1 and NBTXR3 + PRT + αPD1 led to significantly fewer lung metastases compared to XRT + αPD1 and PRT + αPD1, respectively. NBTXR3 + PRT + αPD1 significantly curtailed the number of lung metastases compared to NBTXR3 + XRT + αPD1. However, there was no significant difference observed in lung metastases count between PRT + αPD1 and XRT + αPD1 groups.

The results demonstrate that combining PRT with αPD1 and NBTXR3 provides the most effective control over tumor growth and significantly extends survival, highlighting its potential as a powerful therapeutic strategy against αPD1-resistant lung cancer.

### Divergent patterns of immune cell Infiltration in tumors induced by proton IRT versus photon IRT

Considering that both XRT and PRT administered comparable total radiation doses, it was hypothesized that the improved tumor control observed with PRT + αPD1 and NBTXR3 + PRT + αPD1, in comparison to XRT + αPD1 and NBTXR3 + XRT + αPD1, could be ascribed to their augmented antitumor immune responses. To further investigate this hypothesis, both irradiated and unirradiated tumors were collected eight days after radiation, and the TIME was analyzed using scRNAseq.

**Fig. 2:**
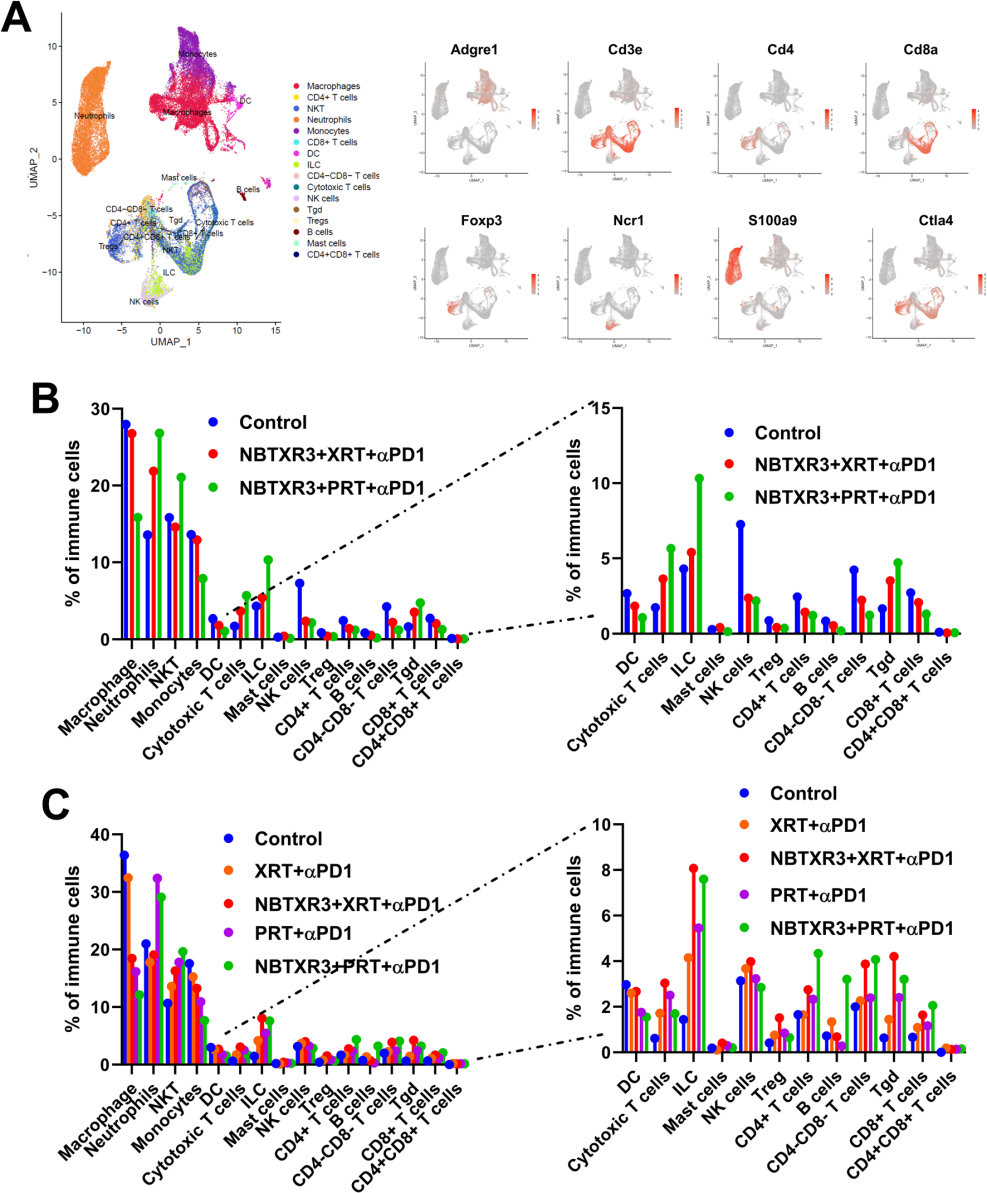
Contrasting immune cell infiltration patterns in tumors induced by proton IRT versus photon IRT. **A** UMAP illustrating immune cell clusters and key markers used to define different immune cell populations. **B** Proportions of different immune cell populations in total immune cells within the irradiated tumors. **C** Proportions of different immune cell populations in total immune cells within the unirradiated tumors. Female 129 Sv/Ev mice aged 8–12 weeks (n = 5) were subjected to varying combinations of NBTXR3, photon radiotherapy, proton radiotherapy, and αPD1 as outlined in Fig. 1A. Primary tumors were harvested 8 days post-radiotherapy from irradiated tumors in groups of Control, NBTXR3 + XRT + αPD1, and NBTXR3 + PRT + αPD1 and unirradiated tumors in groups of Control, XRT + αPD1, NBTXR3 + XRT + αPD1, PRT + αPD1, and NBTXR3 + PRT + αPD1. The immune cell populations within the tumors were examined via scRNAseq

As shown in Fig. 2A, we identified 16 primary immune cell types including macrophages, CD4 + T cells, NKT cells, dendritic cells (DCs), neutrophils, CD8 + T cells, and Tregs using markers such as Adgre1, Cd3e, Cd4, and Cd8a. In irradiated tumors, NBTXR3 + PRT + αPD1 enhanced the infiltration of TILs compared to NBTXR3 + XRT + αPD1. Notable increases included NKT cells (44.38%), cytotoxic T cells (55.80%), ILCs (91.41%), and gamma-delta T cells (33.93%) (Fig. 2B and Supplemental Fig. 2A). Both NBTXR3 treatments reduced Tregs in irradiated tumors, with reductions of 57.11% for NBTXR3 + PRT + αPD1 and 51.28% for NBTXR3 + XRT + αPD1. NBTXR3 + PRT + αPD1 led to decreases in macrophages (40.86%), DCs (41.96%), and monocytes (38.98%), alongside a notable increase in neutrophils (22.65%).

In unirradiated tumors, as depicted in Fig. 2C and Supplemental Fig. 2B, PRT + αPD1 altered the immune cell landscape compared to XRT + αPD1. It reduced the infiltration of macrophages by 50.27%, monocytes by 28.47%, DCs by 32.53%, and B cells by 79.44%, while increasing neutrophils by 82.40%, NKT cells by 30.84%, cytotoxic T cells by 46.30%, ILCs by 31.77%, CD4 + T cells by 41.48%, and Tgd cells by 66.68%.

In a similar pattern to irradiated tumors, NBTXR3 + PRT + αPD1 reduced the infiltration of macrophages by 34.40%, monocytes by 42.31%, and DCs by 42.28%, but increased NKT cells by 20.60% compared to NBTXR3 + XRT + αPD1 (Fig. 2C). Interestingly, this treatment combination also decreased the presence of cytotoxic T cells by 44.26%, NK cells by 28.47%, and Tgd cells by 23.77%, while boosting neutrophils by 52.53%, CD4 + T cells by 58.00%, and B cells by 369.77%. Notably, NBTXR3 + PRT + αPD1 also reduced the proportion of Tregs by 57.38% relative to NBTXR3 + XRT + αPD1 in these tumors.

Our results revealed a distinct variation in the CD8/Treg ratio between NBTXR3 + PRT + αPD1 and NBTXR3 + XRT + αPD1 treatments. NBTXR3 + PRT + αPD1 manifested a markedly enhanced CD8/Treg ratio in both irradiated and unirradiated tumor settings, as elucidated (Supplemental Fig. 3). While NBTXR3 + XRT + αPD1 exhibited an increase in the CD8/Treg ratio in irradiated tumors relative to the control, NBTXR3 + PRT + αPD1 distinctly demonstrated a heightened CD8/Treg ratio in both irradiated and unirradiated tumor scenarios when compared with the control.

**Fig. 3:**
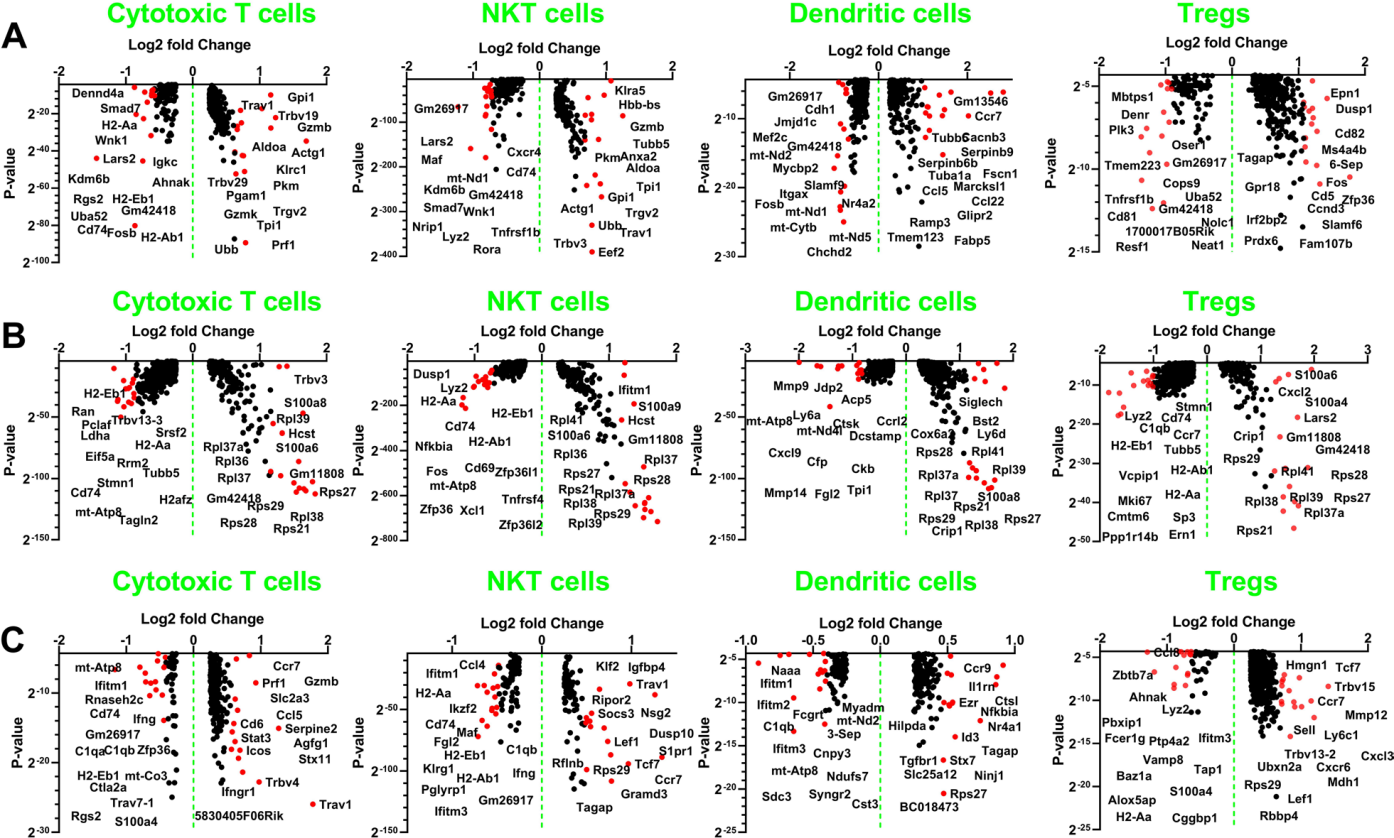
Proton IRT enhances antitumor immune response via favorable gene expression modulation. **A** Differentially expressed genes across various immune cell populations following treatment with NBTXR3 + PRT + αPD1 versus NBTXR3 + XRT + αPD1 in the irradiated tumors. **B** Differentially expressed genes across various immune cell populations following treatment with PRT + αPD1 versus XRT + αPD1 in the unirradiated tumors. **C** Differentially expressed genes across various immune cell populations following treatment with NBTXR3 + PRT + αPD1 versus NBTXR3 + XRT + αPD1 in the unirradiated tumors. Immune cells (CD45 +) were isolated from the tumors exposed to various treatments and examined using scRNAseq. Differentially expressed genes were evaluated using the Kruskal–Wallis test. The top 15 upregulated and downregulated genes are signified by red dots

These results suggest that NBTXR3 + PRT + αPD1 is more effective in modulating the immune landscape to favor antitumor immunity than NBTXR3 + XRT + αPD1, likely contributing to its superior tumor control.

### NBTXR3 combined with proton IRT enhances antitumor immune response through favorable modulation of gene expression

Next, we delved further into understanding the intrinsic mechanisms responsible for the enhancement of immune response through PRT + αPD1 and NBTXR3 + PRT + αPD1. Our examination was particularly focused on the top 15 genes that were significantly upregulated across various immune populations.

Within the irradiated tumors, a significant upregulation was observed in essential genes of the glycolysis pathway such as Gpi1 (Glucose-6-Phosphate Isomerase 1) [25], Tpi1 (Triosephosphate Isomerase 1) [26], Aldoa (Aldolase A) [27], and Pkm (Pyruvate Kinase M) [28] with NBTXR3 + PRT + αPD1 treatment in comparison to NBTXR3 + XRT + αPD1. This surge in gene expression was distinctly notable in cytotoxic T cells and NKT cells (Fig. 3A, Supplemental Fig. 4). Moreover, NBTXR3 + PRT + αPD1 also led to an augmented expression of T cell activation markers, notably Gzmb (Granzyme B) [29], and T cell receptor genes, such as Trgv2 (T Cell Receptor Gamma Variable 2), in conjunction with markers indicative of ubiquitination, particularly Ubb (Ubiquitin B). Within DCs, NBTXR3 + PRT + αPD1 also enhanced the expression of chemokine ligands, such as Ccr7 (C–C Motif Chemokine Receptor 7), Ccl5 (C–C Motif Chemokine Ligand 5), and Ccl22 (C–C Motif Chemokine Ligand 22), as well as genes involved in cellular motility, including Fscn1 (Fascin Actin-Bundling Protein 1) [30], and Marcksl1 (Myristoylated Alanine-Rich Protein Kinase C Substrate-Like 1) [31]. Moreover, in Tregs, we discerned a significant upregulation of Zfp36 (Tristetraprolin) [32], Dusp1 (Dual-Specificity Phosphatase 1) [33], and Ccnd3 (Cyclin D3) [34] under the treatment with NBTXR3 + PRT + αPD1. These changes suggest a possible activation in the population of Tregs and the subsequent enhancement of their suppressive capabilities.

**Fig. 4:**
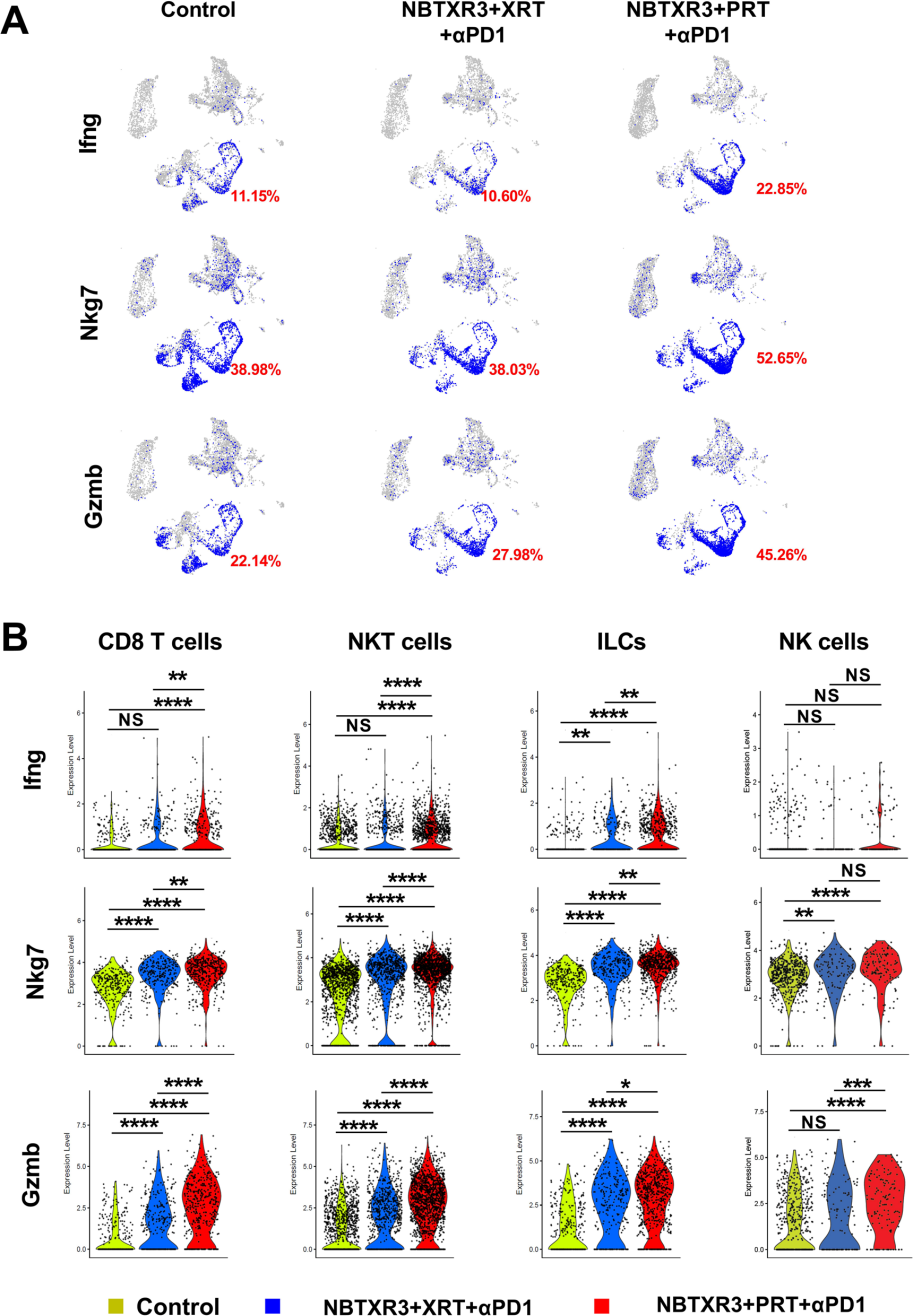
Enhanced promotion of antitumor lymphocyte activation in irradiated tumors by NBTXR3 + PRT + αPD1 Treatment. **A** UMAP color-coded for lymphocyte activation markers. **B** The expression level of antitumor lymphocyte activation markers in cytotoxic lymphocytes. The expression levels of activation markers were assessed using the Kruskal–Wallis test. A p-value of < 0.05 was considered to indicate statistical significance. *P < 0.05; **P < 0.01; ***P < 0.001; ****P < 0.0001. NS, not significant

In addition, when gene expression of NBTXR3 + XRT + αPD1-treated tumors is compared to the control group, there was a notable upregulation of genes such as Ccl5 [35], Ly6a (Lymphocyte Antigen 6 Complex, Locus A) [36], Gzmb, Klrd1 (Killer Cell Lectin-Like Receptor Subfamily D, Member 1) [37], and Icos (Inducible T Cell Co-Stimulator) [38] in cytotoxic T cells (Supplemental Fig. 5A). These genes play crucial roles in either T cell infiltration or activation, with observations made against a control baseline. A similar trend of upregulation was identified for Ccl5, Ly6a, and Icos when comparing NBTXR3 + PRT + αPD1 with control for the same population of cells (Supplemental Fig. 5B).

**Fig. 5:**
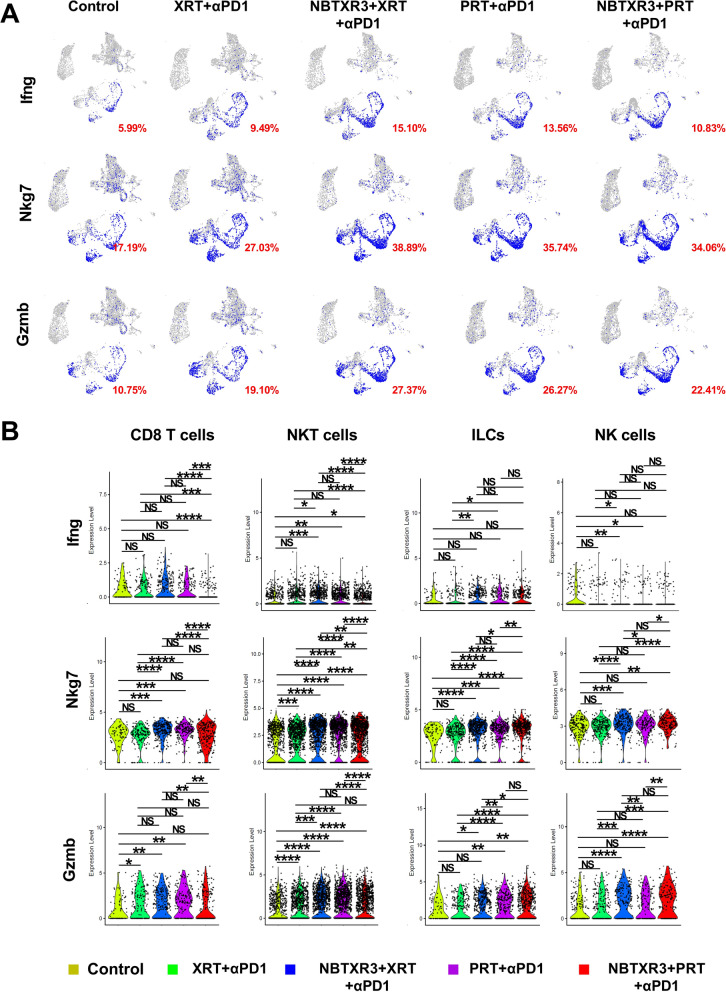
Augmented antitumor lymphocyte activation in unirradiated tumors by PRT + αPD1 compared to XRT + αPD1. **A** UMAP color-coded for lymphocyte activation markers in unirradiated tumors subjected to various combination therapies involving NBTXR3, XRT, PRT, and αPD1. **B** The expression level of antitumor lymphocyte activation markers in cytotoxic lymphocytes. The expression levels of activation markers were analyzed using the Kruskal–Wallis test. A p-value of < 0.05 was deemed statistically significant. *P < 0.05; **P < 0.01; ***P < 0.001; ****P < 0.0001. NS, not significant

In the unirradiated tumor, PRT + αPD1 exhibited a significant upregulation of genes encoding ribosomal proteins including Rpl34, Rpl36, Rpl37, Rpl39, Rpl37a, Rpl38, Rps29, Rps28, Rps27, Rps21, and Trbv3 (T Cell Receptor Beta Variable 3), as compared to XRT + αPD1 (Fig. 3B), for cytotoxic T cells, NKT cells, DCs and Treg.

Still in the untreated tumor, when compared to NBTXR3 + XRT + αPD1, NBTXR3 + PRT + αPD1 treatment resulted in a significant increase in the expression of activation markers such as Gzmb, Icos, Prf1 (Perforin 1) [39], as well as chemokine receptor (Ccr7) [40], and chemokine ligand (Ccl5) in cytotoxic T cells (Fig. 3C). NBTXR3 + PRT + αPD1 also exhibited an increased expression of Ctsl (Cathepsin) [41], which is implicated in antigen presentation and cross-presentation to T cells, Ninj1 (Ninjurin-1)[42] and Ccr9 (C–C Chemokine Receptor Type 9) [43], both integral to DC migration. Furthermore, NBTXR3 + PRT + αPD1 treatment led to increased expression of Ccr7, Cxcl3 (C-X-C Motif Chemokine Ligand 3) [44], Cxcr6 (C-X-C Chemokine Receptor Type 6) [45], which are involved in Treg migration and localization, and T cell receptor genes, potentially contributing to Treg clonal expansion and activation (Fig. 3C).

In comparison to the secondary tumor of control group, there was a significant upregulation of various genes under different treatments. Notably, XRT + αPD1 significantly increased the expression of Gzma (Granzyme A) [46], Ly6a, and Trbv13-3 (T Cell Receptor Beta Variable 13–3) in cytotoxic T cells (Supplemental Fig. 5C). Under NBTXR3 + XRT + αPD1, there was a significant upregulation of Ccl3 (Chemokine (C–C Motif) Ligand 3), Ly6a, Ccl4 (Chemokine (C–C Motif) Ligand 4), and Ccl5 in cytotoxic T cells (Supplemental Fig. 5D).

We noted that PRT + αPD1 significantly upregulated the expression of genes including Lars2, Ly6a, Ccl5, Trav6-3, Trbv12-2, among others, relative to the control group in cytotoxic T cells (Supplemental Fig. 5E). Furthermore, NBTXR3 + PRT + αPD1 significantly amplified the expression of Gzma, Gzmb, Icos, Ly6a, and Prf1, among other genes, in comparison to the control in cytotoxic T cells (Supplemental Fig. 5F).

Finally, we scrutinized the differences in gene expression in between irradiated and unirradiated tumors that were treated with NBTXR3 + XRT + αPD1 and NBTXR3 + PRT + αPD1. In the irradiated tumors treated with NBTXR3 + XRT + αPD1, there was a marked increase in the expression of Arg1 (Arginase 1), a gene that modulates nitric oxide for antitumor activity [47]. We also found an upregulation of Ifngr1 (Interferon Gamma Receptor 1) [48], Icos, and Hif1a (Hypoxia Inducible Factor 1 Subunit Alpha) [49], the latter of which assists cells in adapting to hypoxic conditions in cytotoxic T cells (Supplemental Fig. 6A and 6C). Comparatively, in irradiated tumors treated with NBTXR3 + PRT + αPD1, we observed an increased expression of Trbv19 (T-Cell Receptor Beta Variable 19), Gzmb, Prf1, Ly6a, and Ccl6 (C–C Motif Chemokine Ligand 6) in cytotoxic T cells (Supplemental Fig. 6B and 6D), suggesting that there was an enhancement of T cell functionality in irradiated tumors, which was characterized by a heightened level of activation.

**Fig. 6:**
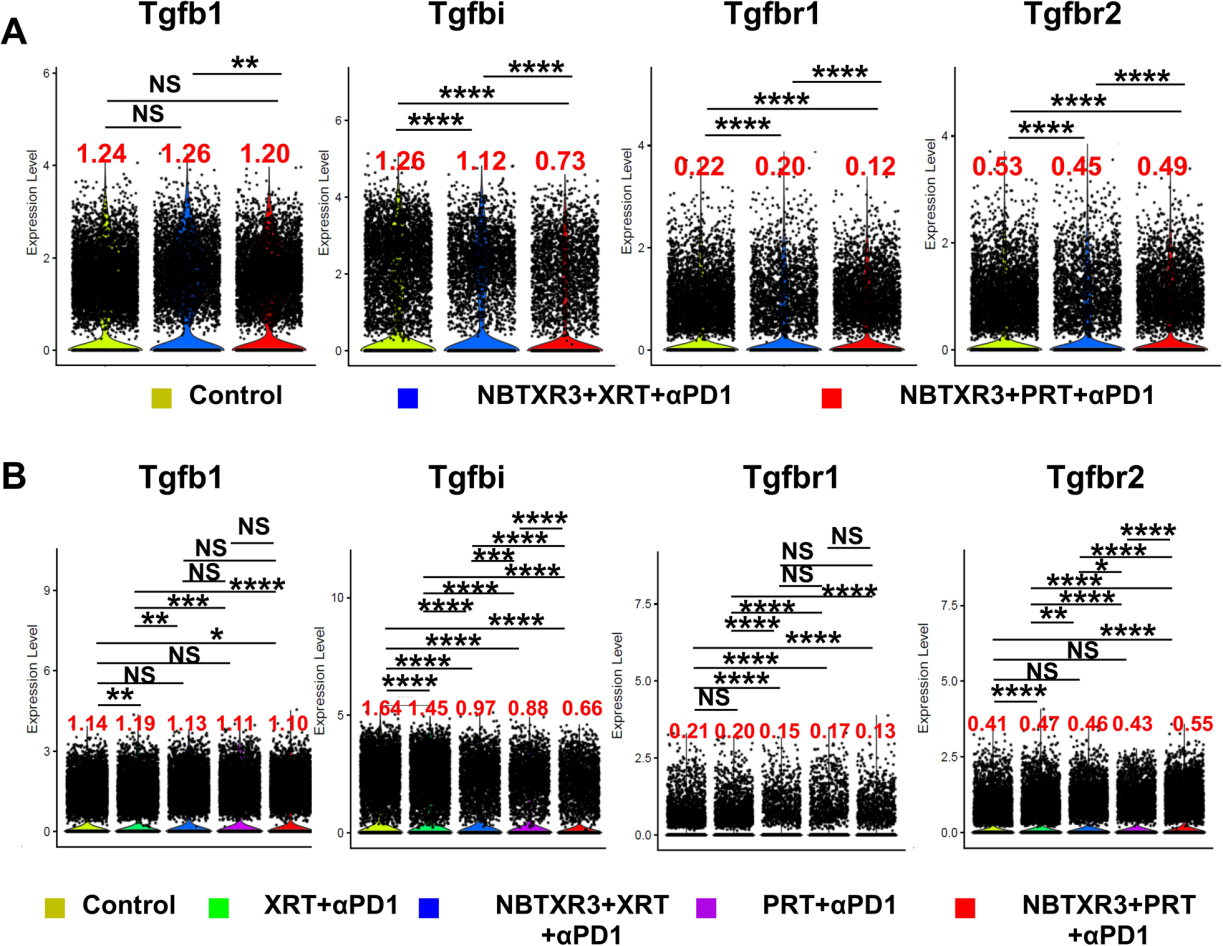
Attenuated expression of genes in the TGF-β pathway by NBTXR3 + PRT + αPD1 compared to NBTXR3 + XRT + αPD1. **A** Expression levels of Tgfb1, Tgfbi, Tgfbr1, and Tgfbr2 in irradiated tumors in the groups: Control, NBTXR3 + XRT + αPD1, and NBTXR3 + PRT + αPD1. **B** Expression levels of Tgfb1, Tgfbi, Tgfbr1, and Tgfbr2 in unirradiated tumors in the groups: Control, XRT + αPD1, NBTXR3 + XRT + αPD1, PRT + αPD1, and NBTXR3 + PRT + αPD1. The expression levels of Tgfb1, Tgfbi, Tgfbr1, and Tgfbr2 were analyzed using the Kruskal–Wallis test. A p-value of < 0.05 was deemed statistically significant. *P < 0.05; **P < 0.01; ***P < 0.001; ****P < 0.0001. NS, not significant

These results collectively demonstrate that NBTXR3 + PRT + αPD1 treatment excels in modulating the immune environment by boosting gene expression related to T cell activation, chemotaxis, and cellular metabolism, resulting in a more potent immune response against tumors.

### Combination of NBTXR3 with PRT + αPD1 demonstrates superior promotion of antitumor lymphocyte activation in irradiated tumors

As depicted in Fig. 3, the application of NBTXR3 + XRT + αPD1 and NBTXR3 + PRT + αPD1 treatment regimens appeared to stimulate lymphocyte activation. To delve deeper into the activation status of antitumor lymphocytes, we compared the expression levels of several activation markers, namely Ifng, Nkg7, Gzmb, Prf1, and Gzma. Within the irradiated tumors, NBTXR3 + PRT + αPD1 drastically elevated the proportions of cells expressing Ifng, Nkg7, Gzmb, Gzma, and Prf1 by 115.57%, 38.44%, 61.76%, 25.63%, and 78.07%, respectively, relative to NBTXR3 + XRT + αPD1 (Fig. 4A and supplemental Fig. 7A).

**Fig. 7:**
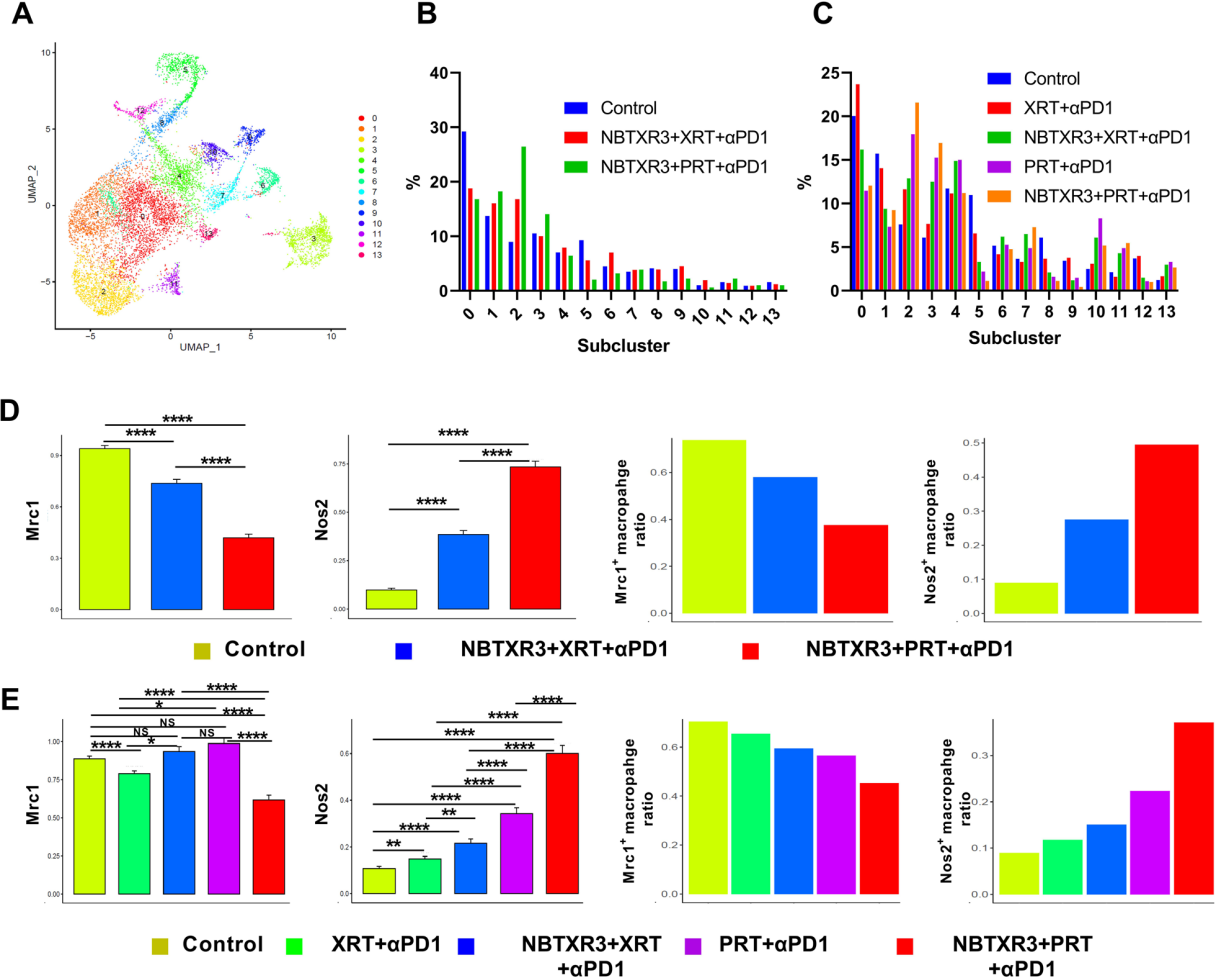
NBTXR3 + PRT + αPD1 treatment promotes a shift toward proinflammatory M1 phenotype in tumor-associated macrophages. **A** UMAP of macrophage subclusters. **B** Proportions of macrophage subclusters in irradiated tumors. **C** Proportions of macrophage subclusters in unirradiated tumors. **D** Expression of Mrc1 and Nos2 in macrophages within irradiated tumors in the groups: Control, NBTXR3 + XRT + αPD1, and NBTXR3 + PRT + αPD1. **E** Expression of Mrc1 and Nos2 in macrophages within unirradiated tumors in the groups: Control, XRT + αPD1, NBTXR3 + XRT + αPD1, PRT + αPD1, and NBTXR3 + PRT + αPD1. The expression levels of Mrc1 and Nos2 were analyzed using the Kruskal–Wallis test. A p-value of < 0.05 was deemed statistically significant. *P < 0.05; **P < 0.01; ***P < 0.001; ****P < 0.0001. NS, not significant

Subsequently, we examined in the treated tumors the expression levels of these activation markers across various lymphocytes, including CD8 T cells, NKT cells, ILCs, and NK cells (Fig. 4B and supplemental Fig. 7B). NBTXR3 + XRT + αPD1 significantly enhanced the expression of Ifng in ILCs, Nkg7 in all lymphocytes, Gzmb and Prf1 in all lymphocytes, excluding NK cells. NBTXR3 + PRT + αPD1 substantially increased the expression of all markers except Gzma in lymphocytes and Ifng in NK cells. Notably, relative to NBTXR3 + XRT + αPD1 in the primary tumors, NBTXR3 + PRT + αPD1 induced a significantly higher expression of activation markers, except Gzma in lymphocytes.

Additionally, we analyzed the expression of lymphocyte activation markers in the unirradiated tumors across different treatment groups (Fig. 5A and supplemental Fig. 8A). All treatments substantially augmented the proportion of cells expressing all the activation markers relative to the control. Moreover, when compared to XRT + αPD1, PRT + αPD1 increased the proportion of cells expressing Ifng, Nkg7, Gzmb, and Prf1 by 42.89%, 32.22%, 37.54%, and 55.40%, respectively. However, fewer cells expressing these activation markers were observed in NBTXR3 + PRT + αPD1 compared to NBTXR3 + XRT + αPD1.

**Fig. 8:**
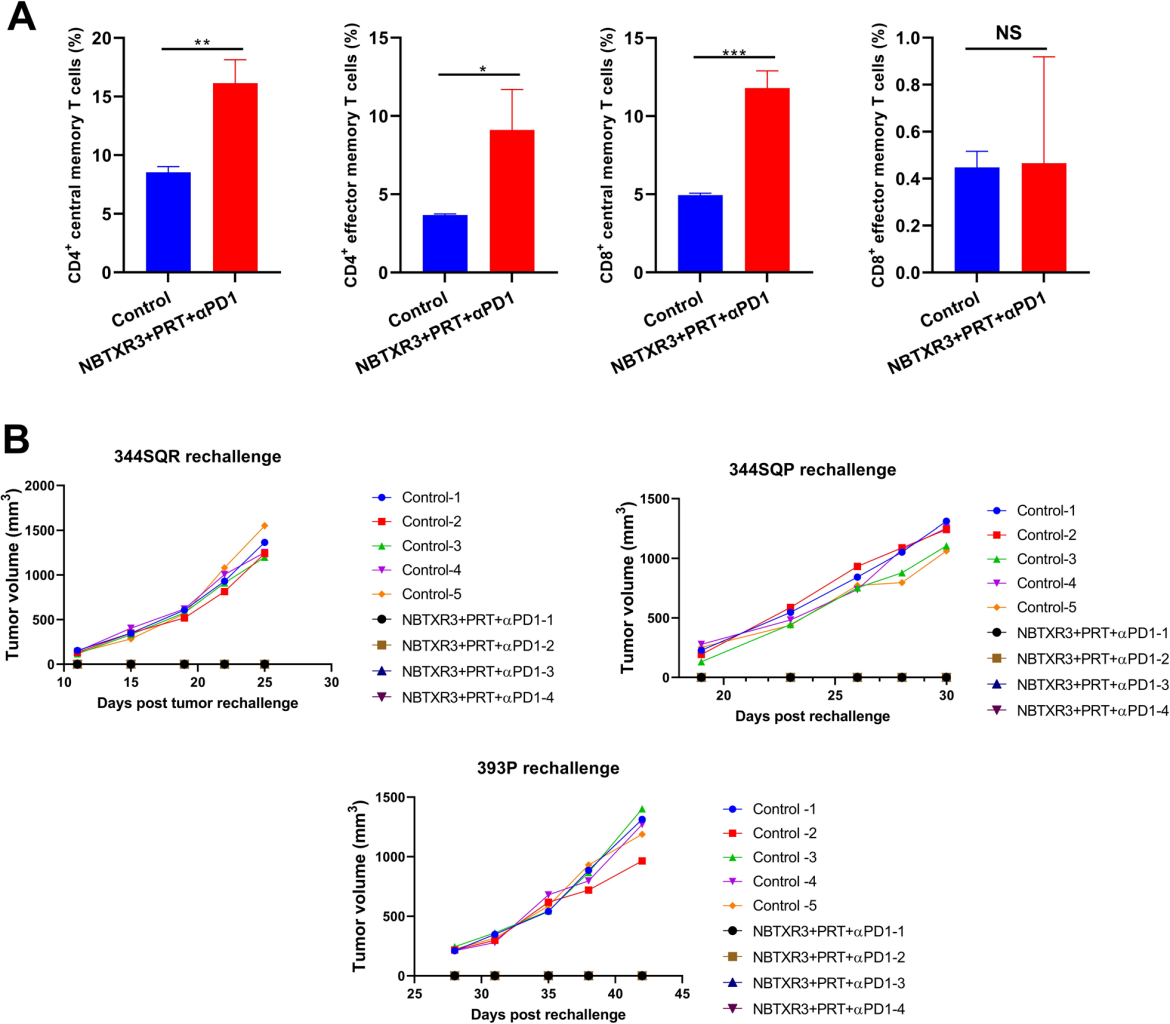
Induction of potent antitumor memory immune response through combination therapy of NBTXR3, PRT, and αPD1. **A** Presents the percentages of CD4 + and CD8 + memory T cells in the blood. **B** Demonstrates the growth curves of tumors in mice that survived and were rechallenged with 344SQR, 344SQP, and 393P cells. Memory T cell populations in the blood of these survivor mice that received NBTXR3 + PRT + αPD1 were characterized 93 days post PRT. These mice were then subjected to a rechallenge with 5X10^4^ 344SQR cells 102 days post PRT, 5X10^5^ 344SQP cells 157 days post PRT, and 5 × 10^5^ 393P cells on 247 days post PRT. Percentages of blood memory T cells were analyzed using two-tailed t-tests. The data are presented as mean ± SEM. The threshold for statistical significance was set at P < 0.05. Indicators of statistical significance are as follows: *P < 0.05; **P < 0.01; ***P < 0.001. NS denotes findings that were not significant

Concerning expression level in untreated tumors, XRT + αPD1 significantly escalated the level of Nkg7 in NKT cells and Gzmb in CD8 T cells and NKT cells relative to the control (Fig. 5B and supplemental Fig. 8B). PRT + αPD1 significantly elevated the expression of Ifng and Gzmb in NKT cells, Nkg7 in CD8 T cells, NKT cells, and ILCs, and Prf1 in NKT cells in comparison to the control. Moreover, PRT + αPD1 induced a significant increase in expression levels of Ifng in ILCs, Nkg7 and Prf1 in CD8 T cells, NKT cells, and ILCs, Gzmb in NKT cells and ILCs, and Gzma in NKT cells compared to XRT + αPD1. NBTXR3 + PRT + αPD1 had higher expression levels of Ifng in NKT cells, Gzmb and Nkg7 in NKT cells, ILCs, and NK cells, Gzma in NK cells, and Prf1 in NKT and NK cells than the control. Unexpectedly, NBTXR3 + PRT + αPD1 demonstrated significantly lower expression levels of Ifng in CD8 T cells and NKT cells, Nkg7 and Gzmb in all lymphocytes excluding NK cells, and Prf1 in NKT cells than NBTXR3 + XRT + αPD1.

Given the potential for persistent lymphocyte activation to culminate in exhaustion, we assessed the expression levels of exhaustion markers, including Pdcd1 (coding for PD1 protein), Havcr2 (coding for TIM-3 protein), Lag3, Tigit, and Ctla4. As depicted in Supplemental Fig. 9A, NBTXR3 + PRT + αPD1 significantly upregulated the expression of these four immune checkpoint receptors (ICRs) within CD4 + T cells, CD8 + T cells, or both in the irradiated tumors compared to the control. A similar pattern was observed with NBTXR3 + XRT + αPD1, which also significantly elevated the expression of these ICRs in CD4 + T cells, CD8 + T cells, or both in the irradiated tumors relative to the control. As anticipated, NBTXR3 + PRT + αPD1 was associated with a notably higher expression of ICRs in the irradiated tumors compared to NBTXR3 + XRT + αPD1.

However, in the unirradiated tumors (Supplemental Fig. 9B), NBTXR3 + PRT + αPD1 demonstrated lower levels of Pdcd1, Lag3, Tigit, and Ctla4 compared to NBTXR3 + XRT + αPD1 in CD4 + T cells, CD8 + T cells, or both. In contrast, PRT + αPD1 resulted in a significantly elevated expression of these exhaustion markers compared to XRT + αPD1 in the unirradiated tumors. These findings corroborate the proposition that increased lymphocyte activation may be accompanied by heightened lymphocyte exhaustion.

These results indicate that while the combination of NBTXR3 with PRT + αPD1 generally enhances lymphocyte activation more effectively than NBTXR3 + XRT + αPD1, it also leads to higher levels of lymphocyte exhaustion, particularly in irradiated tumors.

### Modulation of TGF-β pathway gene expression by PRT + αPD1 and NBTXR3 + PRT + αPD1 treatments

An upregulation in the activity of the transforming growth factor-beta (TGF-β) pathway is commonly noted in tumors following treatment with radiotherapy [50]. The TGF-β pathway is well-acknowledged as a pivotal modulator of the antitumor immune response, typically exhibiting inhibitory effects [51]. Contrastingly, we have observed a more advantageous immune response post PRT + αPD1 and NBTXR3 + PRT + αPD1 as compared to XRT and NBTXR3 + XRT + αPD1. Hence, we hypothesize that the treatments PRT + αPD1 and NBTXR3 + PRT + αPD1 may contribute to the downregulation of TGF-β pathway activity. To validate our hypothesis, we conducted a detailed examination of pivotal genes integral to the TGF-β pathway, specifically Tgfb1 (Transforming Growth Factor Beta 1), Tgfbi (Transforming Growth Factor Beta-Induced), Tgfbr1 (Transforming Growth Factor Beta Receptor 1), and Tgfbr2 (Transforming Growth Factor Beta Receptor 2). Our findings, as illustrated in Fig. 6A and Supplemental Fig. 10A, demonstrate significant modulation of gene expression within the TGF-β pathway in irradiated tumors. Notably, NBTXR3 + PRT + αPD1 resulted in significant reductions in the expression levels of Tgfb1, Tgfbi, Tgfbr1, and Tgfbr2 compared to the control group. NBTXR3 + XRT + αPD1 also led to a decline in the expression of Tgfbi, Tgfbr1, and Tgfbr2 relative to the control. However, the expression levels of Tgfb1, Tgfbi, and Tgfbr1 were significantly lower following NBTXR3 + PRT + αPD1 compared to NBTXR3 + XRT + αPD1.

In unirradiated tumors, XRT + αPD1 significantly increased the expression of Tgfb1 while concurrently reducing the levels of Tgfbi compared to the control group, as shown in Fig. 6B and Supplemental Fig. 10B. In contrast, PRT + αPD1 resulted in expression levels of Tgfb1 and Tgfbr2 that were comparable to the control group but significantly reduced the levels of Tgfbi and Tgfbr1. Furthermore, PRT + αPD1 yielded significantly lower levels of Tgfb1, Tgfbi, Tgfbr1, and Tgfbr2 compared to XRT + αPD1. Interestingly, the addition of NBTXR3 to XRT + αPD1 led to significant reductions in the expression of all four genes in the unirradiated tumors. Compared to NBTXR3 + XRT + αPD1, NBTXR3 + PRT + αPD1 resulted in a significant reduction in the expression of Tgfbi.

Furthermore, we examined the expression of TNFα (Tumor Necrosis Factor-alpha), a cytokine known to engage in significant interplay with TGF-β [52]. TNFα critically contributes to tumor control via several mechanisms: inducing apoptosis in tumor cells; augmenting the antitumor functions of cytotoxic T cells and NK cells; impeding the tumor's vascular supply; and instigating macrophages to generate reactive oxygen species and nitric oxide, which exhibit cytotoxicity towards tumor cells [53]. As demonstrated in Supplemental Fig. 11A, B, NBTXR3 + PRT + αPD1 resulted in a significantly elevated TNFα expression compared to NBTXR3 + XRT + αPD1, evident in both irradiated and unirradiated tumors. Interestingly, in the unirradiated tumors, TNFα levels did not exhibit an exact inverse relationship with TGF-β. This discrepancy may be attributable to the possibility that TNFα generated at the irradiated site may also disseminate to distant tumors.

These results highlight the potential of NBTXR3 + PRT + αPD1 treatment in enhancing antitumor immune responses through the dual modulation of TGF-β and TNFα pathways, suggesting a promising therapeutic strategy for effective cancer treatment.

### Proton IRT facilitates a shift towards proinflammatory M1 phenotype in tumor-associated macrophages

In our preceding study [22], we discovered that both PRT + αPD1 and NBTXR3 + PRT + αPD1 fostered the infiltration of M1 macrophages into both irradiated and unirradiated tumors. As various cancer types exhibit an improved prognosis when tumor-associated macrophages (TAMs) shift from the M2 to the M1 phenotype [54], we further investigated this in the present study. Here, we meticulously delineated the subclusters of macrophages and gauged the impact of XRT and PRT on macrophage polarization.

As shown in Fig. 7A, Supplemental Fig. 12, and Supplemental Fig. 13, we identified 14 macrophage subpopulations according to distinct gene expression. Notably, macrophage populations 2, 3, and 11 (MP2, MP3, MP11) exhibited a marked increased percentage post NBTXR3 + PRT + αPD1 treatment compared to NBTXR3 + XRT + αPD1 in both irradiated and unirradiated tumors, and after PRT + αPD1 compared to XRT + αPD1 in unirradiated tumors (Fig. 7B, C).

Intriguingly, within the MP2 macrophages (Supplemental Fig. 12), we observed a substantial upregulation of glycolysis-related genes such as Pgk1 (Phosphoglycerate Kinase 1), Gapdh (Glyceraldehyde-3-Phosphate Dehydrogenase), Aldoa (Aldolase, Fructose-Bisphosphate A), Slc2a1 (GLUT1), and Gpi1 (Glucose-6-Phosphate Isomerase 1) [55]. The increased expression of these genes within the MP2 population potentially signifies a shift toward M1 macrophages. This aligns with the established understanding that M1 macrophages rely more heavily on glycolysis, highlighting a possible metabolic reprogramming in this population [56].

In the MP3 population (Supplemental Fig. 12), we found increased expression of Cd3d, Cd3e, and Cd3g—typically T cell markers comprising the CD3 complex that, with the T cell receptor (TCR), forms the TCR complex pivotal in T-cell activation. However, recent studies reported that certain macrophages express CD3 [57], and these populations may create proinflammatory environments conducive to enhancing the antitumor immune response.

Conversely, in unirradiated tumors, after NBTXR3 + PRT + αPD1 compared to NBTXR3 + XRT + αPD1 treatment in the two tumors and PRT + αPD1 compared to XRT + αPD1, we noted reduced percentages of MP0 and MP1 (Fig. 7C). MP0 macrophages exhibited elevated expression of Gatm (Glycine Amidinotransferase)[58] and Cxcl16 (C-X-C Motif Chemokine Ligand 16) [59], both associated with M2 macrophage polarization (Supplemental Fig. 12). In the MP1 population (Supplemental Fig. 12), we identified increased expression of Fcrla (Fc Receptor-Like A) [60], Ucp2 (Uncoupling Protein 2, where enhanced UCP2 expression correlates with a higher efferocytosis capacity in macrophages, which suppresses antitumor immunity) [61], Eno1 (Enolase 1) [62], and Fn1 (Fibronectin 1)[63]—all markers correlating with M2 macrophage polarization.

We further evaluated the expression levels of M2 marker (Mrc1, Mannose Receptor C-type 1, as known as CD206) [64] and M1 marker (Nos2, Nitric Oxide Synthase 2, as denoted iNOS) [65] in both irradiated and unirradiated tumors following various combination therapy, as depicted in Fig. 7D, E. Our findings revealed that both NBTXR3 + XRT + αPD1 and NBTXR3 + PRT + αPD1 significantly diminished the expression of Mrc1 while concurrently elevating the expression of Nos2 in macrophages, compared to the control group. Moreover, NBTXR3 + PRT + αPD1 was observed to induce a substantial decrease in Mrc1 expression and a corresponding increase in Nos2 expression compared to NBTXR3 + XRT + αPD1 in the irradiated tumors.

Regarding unirradiated tumors, NBTXR3 + PRT + αPD1 was also found to significantly decrease Mrc1 expression and increase Nos2 expression in relative to NBTXR3 + XRT + αPD1. Additionally, PRT resulted in a significantly higher increase in Nos2 expression compared to XRT in the unirradiated tumors. Our data further revealed that tumors treated with PRT exhibited a decreased proportion of macrophages expressing Mrc1, compared to those treated with XRT. Conversely, a heightened percentage of macrophages demonstrated Nos2 expression in tumors subjected to PRT in comparison to those treated with XRT (Figs. 7D and 7E).

These results suggest that PRT, especially when combined with NBTXR3 and αPD1, promotes a more advantageous antitumor immune response by fostering a shift from M2 to M1 macrophage polarization, which is more favorable for tumor control.

### NBTXR3 + PRT + αPD1 induces a robust antitumor memory immune response and rejection of diverse cancer cells

In prior studies [18, 19, 22, 23, 66], it was consistently documented that mice subjected to IRT exhibited strong antitumor memory immune responses, effectively suppressing tumor formation upon subsequent reintroduction of tumor cells. Such potent memory immunity could potentially enable patients to avert tumor recurrence in clinical settings. As depicted in Fig. 1C, only NBTXR3 + PRT + αPD1 treatment was able to completely destroy both treated and untreated tumors and induced long term survival in 40% of mice. Consequently, we expanded our investigation to examine the populations of memory T cells present in the blood of these surviving mice using flow cytometry (Supplemental Fig. 14).

As demonstrated in Fig. 8 A, mice treated with NBTXR3 + PRT + αPD1 exhibited a significant increase in the percentage of CD4 + central memory T cells (16.15 ± 2.00% vs 8.54 ± 0.47%), CD4 + effector memory T cells (9.11 ± 2.59% vs 3.67 ± 0.07%), and CD8 + central memory T cells (11.79 ± 1.11% vs 4.94 ± 0.12%). Historically, our research only rechallenged the mice with identical cancer cells as those used for assessing the efficacy of treatments [18, 19, 22, 23]. However, in the current study, we extended this to include 344SQR, 344SQP, and 393P cell lines. These cell lines represent different but closely related forms of lung adenocarcinoma. Specifically, 393P and 344SQP represent primary non-metastatic and subcutaneous metastatic murine lung adenocarcinoma cell lines, respectively [24]. 344SQR is an αPD1-resistant lung adenocarcinoma cell line derived from 344SQP, which is αPD1-sensitive [16]. The goal of this expansion was to investigate the potential for these mice to also inhibit tumor growth when exposed to these closely related cell lines. As depicted in Fig. 8B, the four mice that survived in the NBTXR3 + PRT + αPD1 group not only rejected the establishment of the original 344SQR cell tumors, but also inhibited tumor growth in the case of 344SQP and 393P cells. Our research observations indicate that the surviving mice demonstrated a potent immunological memory response.

These results highlight the potential of combining PRT with NBTXR3 and αPD1 in creating a strong and lasting immune memory that can effectively counteract multiple forms of lung adenocarcinoma.

## Discussion

XRT has remained the most widely utilized form of radiotherapy over the past few decades. Conversely, the first clinical application of PRT was initiated to treat patients at the Berkeley Radiation Laboratory at the University of California in 1954 [67]. Its adoption, however, has been limited due to substantial costs and logistical complexities [1]. Recently, in response to a growing demand for high-precision and safer treatment options, the use of PRT has seen a rapid expansion [68].

With the emergence of immunotherapy, achieving an abscopal effect—systemic disease control extending beyond localized radiation treatment—is now a realistic possibility in patients undergoing combination therapy of radiotherapy and immune checkpoint inhibitors (ICIs) [69]. Yet, most IRT studies are based on XRT, and reports of IRT involving PRT are sparse. PRT might offer unique advantages in augmenting antitumor immune responses. Its capacity for precision in delivering radiation energy to tumors, while avoiding collateral damage to surrounding lymphoid tissues and immune cells within blood vessels, can effectively mitigate lymphopenia [70]. Consequently, PRT presents a potentially advantageous strategy for enhancing the effectiveness of immunotherapies by ensuring the preservation and functionality of the immune system's cellular constituents.

Our recent investigations revealed that both XRT and PRT, when used in conjunction with ICIs, could enhance the antitumor immune response in both irradiated and unirradiated tumors, significantly prolonging survival in murine models [8, 22, 23]. Conducting a comparative analysis to evaluate the efficacy of IRT based on XRT and PRT would be a compelling pursuit. The outcomes from such comparative study could offer critical insights that may significantly inform the clinical application of these combination therapies. Our findings indicated that PRT + αPD1 exhibited notably superior efficacy in controlling both locally irradiated tumors and distant unirradiated metastases, compared to its XRT + αPD1 counterpart. This study, for the first time, demonstrates that PRT-based IRT yields a significantly more robust antitumor response compared to XRT-based IRT, highlighting the potential superiority of PRT in the immunoradiotherapeutic context. The higher LET of PRT may lead to a more effective activation of the antitumor immune response compared to XRT. This is supported by previous studies indicating that radiation with high LET can more efficiently initiate the production of IFN-beta, a key factor in immune response, than radiation with low LET [71, 72].

Previous research has established that NBTXR3 nanoparticles are biologically inert and exhibit a favorable safety profile in clinical settings [73–75]. It is noteworthy to underscore that the addition of NBTXR3 significantly enhanced the efficacy of both XRT + αPD1 and PRT + αPD1; however, the antitumor effectiveness of NBTXR3 + PRT + αPD1 remained significantly higher than that of NBTXR3 + XRT + αPD1. The alterations observed within the TIME can be attributed to the radiation-enhancing effects of NBTXR3, leading to an amplified therapeutic impact.

Through an extensive analysis of the TIME using scRNAseq, we discovered that PRT + αPD1 has a greater capacity to modulate the infiltration of TILs into both irradiated and unirradiated tumors compared to XRT + αPD1. This observation could substantiate the superior treatment outcomes associated with PRT + αPD1, which might be attributed to enhanced infiltration of lymphocytes into the tumor. Our research, along with that of other investigators, suggests that an increased presence of TILs often correlates with the improved therapeutic efficacy of IRT [8, 22, 23, 76, 77].

Notably, the diminished presence of Tregs in the unirradiated tumors within NBTXR3 + PRT + αPD1 group, compared to NBTXR3 + XRT + αPD1 group, could significantly mitigate the immune suppressive effects exerted by Tregs [78]. The pronounced increase in the CD8/Treg ratio, as seen in both the irradiated and unirradiated tumors within the NBTXR3 + PRT + αPD1 group, suggests a dual phenomenon: a reduction in immune suppression and an enhancement of antitumor immunity. These findings could provide a partial rationale for the superior therapeutic efficacy observed with NBTXR3 + PRT + αPD1 [79, 80]. Despite these favorable changes in immune populations, we observed that the upregulated expression of Ccr7, Cxcl3, and Cxcr6, which are essential for Treg migration, contrasts with the reduced presence of Tregs in unirradiated tumors. Similarly, the upregulated expression of Ninj1 and Ccr9 in DCs, which contribute to their migration, is paradoxically accompanied by a reduced DC percentage in these tumors. It is possible that the tumor microenvironment downregulates corresponding ligands in response to NBTXR3 + PRT + αPD1 treatment, preventing effective Treg homing to the tumor site. This could explain the reduced Treg presence despite increased expression of migration-associated genes. Likewise, the decreased DC proportions, despite the upregulation of migration-related genes, suggest a complex interplay where the tumor microenvironment may actively resist DC infiltration and function. Additionally, DCs might be redirected to nearby draining lymph nodes for tumor antigen presentation to T cells. These intriguing discrepancies underscore the need for approaches like spatial genomics to analyze the spatial distribution and phenotypic profiles of immune cells within the tumor microenvironment, offering deeper insights into how treatments affect cell–cell interactions and microenvironmental dynamics.

It is noteworthy that NBTXR3 + PRT + αPD1 led to a decreased population of CD8 T cells and macrophages compared to the control group. Interestingly, a previous study observed that this combination therapy had an opposing effect on these two cell populations [23]. This discrepancy may be attributable to variations in the timing of tumor harvest between the studies.

Furthermore, in addition to the quantity of antitumor lymphocytes, the augmented expression of cytotoxic lymphocyte activation markers—specifically Granzyme B and Perforin —in PRT + αPD1-treated tumors compared to XRT + αPD1-treated tumors indicates that PRT + αPD1 possesses a superior capacity for antitumor immune activation. Perforin and Granzyme B play critical roles in the antitumor function of lymphocytes. Perforin first forms pores in the tumor cell membrane, followed by Granzyme B triggering an apoptosis cascade, thus implementing their cytotoxic effects [39].

The diminished functionality of TGF-β pathways, evidenced by the downregulated expression of Tgfb1, Tgfbr1, and Tgfbr2 in tumors treated with PRT + αPD1 versus XRT + αPD1, and NBTXR3 + PRT + αPD1 versus NBTXR3 + XRT + αPD1, may partially account for the enhanced antitumor activity observed in PRT + αPD1-treated tumors. TGF-β is ubiquitously expressed across a variety of immune cell types, including T cells, B cells, macrophages, and dendritic cells [81]. It exerts substantial inhibitory effects on the proliferation and activation of T cells, NK cells, and B cells. Moreover, TGF-β can facilitate the polarization of immunosuppressive M2 macrophages and augment the differentiation of Tregs. Therefore, the attenuated expression of TGF-β and its associated receptors induced by IRT could significantly alleviate antitumor suppression and promote immune activation, thereby augmenting the efficacy of the antitumor response. In contrast to the control group, tumors treated with NBTXR3 + PRT + αPD1 exhibited significantly elevated levels of TNF-α expression. This increased expression of TNF-α could be a direct consequence of reduced TGF-β expression observed within the NBTXR3 + PRT + αPD1 group [52]. An increase in TNF-α levels could contribute, in a nonspecific manner, to antitumor activities in both irradiated and non-irradiated tumors.

M1 macrophages, known for their antitumor properties, can produce proinflammatory cytokines such as IL-12 and TNF-α, as well as oxygen intermediates, all of which are crucial in countering cancer [82, 83]. Additionally, M1 macrophages exhibit high expression levels of MHC class II molecules and co-stimulatory molecules, including CD86 and CD80, that are vital for T cell activation [84, 85]. On the other hand, M2 macrophages, which express lower levels of MHC class II molecules and co-stimulatory molecules, secrete anti-inflammatory cytokines such as IL-10 and TGF-β [86]. These cytokines can suppress inflammatory responses, encourage angiogenesis, and promote tumor growth. Our results suggest that PRT + αPD1 can enhance the infiltration of proinflammatory M1 macrophages and reduce the presence of M2 macrophages in both irradiated and unirradiated tumors. Additionally, both PRT + αPD1 and NBTXR3 + PRT + αPD1 significantly shift the macrophage population toward the M1 phenotype, a change that could be partially attributed to the inhibition of the TGF-β pathways triggered by PRT [87]. This robust M1 polarization effect induced by PRT + αPD1 suggests that PRT + αPD1 can enhance antitumor responses by altering the phenotypes of macrophages.

In the final analysis, we found that mice cured by NBTXR3 + PRT + αPD1 maintained high levels of memory T cells, including CD4 + central and effector memory T cells and CD8 + central memory T cells. These memory lymphocytes can rapidly respond upon re-encountering their specific tumor antigens, delivering a swift and robust antitumor immune response [88]. The inhibited growth of re-challenged 344SQR tumors suggests that memory immunity in cured mice could effectively prevent tumor relapse. Beyond the 344SQR cells, the survivor mice were also capable of rejecting the establishment of tumors by 344SQP and 393P tumor cells. In our previous work [22], we discovered that mice cured through NBTXR3 + PRT + αPD1 treatment not only exhibited an upregulated adaptive memory immune response, but also demonstrated elevated activities in innate immune pathways. It is plausible that the observed rejection of both 344SQP and 393P cells could be attributed to the combined effects of both adaptive and innate memory responses. These findings are of great significance, as many cancer patients in clinical settings will experience tumor relapse of the original tumor or closely related tumor mutants at a certain point after the initial cure [89]. The combination of PRT + αPD1 with NBTXR3 offers the potential to provide lasting protection against tumor relapse.

It is imperative to acknowledge that our study concentrated on comparing the therapeutic efficiency of PRT + αPD1 and XRT + αPD1 in αPD1-resistant lung tumors, as most lung cancer patients exhibit resistance to anti-PD1 therapy [90]. Our future research will explore these differences in other tumor models.

## Conclusions

In summary, our findings underscore the enhanced tumor control and prolonged survival conferred by PRT-based IRT in comparison to XRT. This superiority is underpinned by a more potent local and systemic antitumor immune reaction. Importantly, the synergistic effect of combining NBTXR3 with either PRT + αPD1 or XRT + αPD1 substantially augments the therapeutic outcomes of both modalities. The heightened efficacy is linked to an intensified antitumor immune response. These results offer valuable insights for the clinical translation of XRT and PRT-based IRT, potentially broadening the therapeutic applications of NBTXR3 beyond its current indications.

## Supplementary Information

Supplementary material 1. Fig. 1. Individual tumor growth in irradiated and unirradiated tumors subjected to diverse combination therapies involving NBTXR3, XRT, PRT, and αPD1. Fig. 2. UMPAs of immune cell populations in tumors subjected to different combination therapies of NBTXR3, XRT, PRT, and αPD1. (A) Irradiated tumors. (B) Unirradiated tumors. Fig. 3. CD8/Treg ratio. (A) Irradiated tumors. (B) Unirradiated tumors. Fig. 4. Heatmap illustrating variations in gene expression across diverse immune cells. (A) Differentiated gene expression in irradiated tumors treated with the NBTXR3+PRT+αPD1 combination to those treated with the NBTXR3+XRT+αPD1 combination. (B) Differentiated gene expression in unirradiated tumors subjected to NBTXR3+PRT+αPD1 versus NBTXR3+XRT+αPD1 treatments. (C) Differentiated gene expression in unirradiated tumors treated with PRT+αPD1 versus XRT+αPD1. Fig. 5. Differential gene expression in immune cells across various IRTs. (A) Irradiated tumors under NBTXR3+XRT+αPD1 versus control. (B) Irradiated tumors under NBTXR3+PRT+αPD1 versus control. (C) Unirradiated tumors under XRT+αPD1 versus control. (D) Unirradiated tumors under NBTXR3+XRT+αPD1 versus control. (E) Unirradiated tumors under PRT+αPD1 versus control. (F) Unirradiated tumors under NBTXR3+PRT+αPD1 versus control. Each panel focuses on Cytotoxic T Cells, NKT Cells, Dendritic Cells, and Tregs. The top 15 upregulated and downregulated genes are signified by red dots. Fig. 6. Comparison of gene expression in immune cells between irradiated and unirradiated Tumors. (A) Heatmap showing gene expression differences in irradiated versus unirradiated tumors treated with NBTXR3+XRT+αPD1. (B) Heatmap indicating differential gene expression in irradiated versus unirradiated tumors subjected to NBTXR3+PRT+αPD1. (C) Differential gene expression in immune cells from irradiated versus unirradiated tumors treated with NBTXR3+XRT+αPD1. (D) Differential gene expression in immune cells in irradiated versus unirradiated tumors treated with NBTXR3+PRT+αPD1. The top 15 upregulated and downregulated genes are signified by red dots. Fig. 7. Gzma and Prf1 expression in irradiated tumors. (A) UMAP of Gzma and Prf1 expression within irradiated tumors. (B) Gzma and Prf1 expression levels in irradiated tumors across the following groups: Control, NBTXR3+XRT+αPD1, and NBTXR3+PRT+αPD1. Fig. 8. Gzma and Prf1 expression in unirradiated tumors. (A) UMAP of Gzma and Prf1 expression within unirradiated tumors. (B) Gzma and Prf1 expression levels in unirradiated tumors across the following groups: Control, XRT+αPD1, NBTXR3+XRT+αPD1, PRT+αPD1, and NBTXR3+PRT+αPD1. Fig. 9. Analysis of checkpoint receptor expression in CD4 and CD8 T Cells. (A) Irradiated tumors. (B) Unirradiated tumors. Fig. 10. UMAP visualization of Tgfb1 and Tgfbi expression. (A) Irradiated tumors. (B) Unirradiated tumors. Fig. 11. Analysis of TNFα expression. (A) Irradiated tumors. (B) Unirradiated tumors. Fig. 12. Heatmap of macrophage clustering. Fig. 13. UMAPs of macrophage subclusters across different treatments. (A) Irradiated tumors. (B) Unirradiated tumors. Fig. 14. Flow cytometry gating strategy for identifying memory T cells.

## Data Availability

The data and materials that support the findings of this study are available from the corresponding author, upon reasonable request.
