# Supplementary material for: Superior antitumor immune response achieved with proton over photon immunoradiotherapy is amplified by the nanoradioenhancer NBTXR3
Source: J Nanobiotechnology. 2024 Oct 1;22:597. doi: 10.1186/s12951-024-02855-0 (PMC11445951; doi:10.1186/s12951-024-02855-0)
Supplement: Supplementary file 1 — Supplementary material 1. Fig. 1. Individual tumor growth in irradiated and unirradiated tumors subjected to diverse combination therapies involving NBTXR3, XRT, PRT, and αPD1. Fig. 2. UMPAs of immune cell populations in tumors subjected to different combination therapies of NBTXR3, XRT, PRT, and αPD1. (A) Irradiated tumors. (B) Unirradiated tumors. Fig. 3. CD8/Treg ratio. (A) Irradiated tumors. (B) Unirradiated tumors. Fig. 4. Heatmap illustrating variations in gene expression across diverse immune cells. (A) Differentiated gene expression in irradiated tumors treated with the NBTXR3+PRT+αPD1 combination to those treated with the NBTXR3+XRT+αPD1 combination. (B) Differentiated gene expression in unirradiated tumors subjected to NBTXR3+PRT+αPD1 versus NBTXR3+XRT+αPD1 treatments. (C) Differentiated gene expression in unirradiated tumors treated with PRT+αPD1 versus XRT+αPD1. Fig. 5. Differential gene expression in immune cells across various IRTs. (A) Irradiated tumors under NBTXR3+XRT+αPD1 versus control. (B) Irradiated tumors under NBTXR3+PRT+αPD1 versus control. (C) Unirradiated tumors under XRT+αPD1 versus control. (D) Unirradiated tumors under NBTXR3+XRT+αPD1 versus control. (E) Unirradiated tumors under PRT+αPD1 versus control. (F) Unirradiated tumors under NBTXR3+PRT+αPD1 versus control. Each panel focuses on Cytotoxic T Cells, NKT Cells, Dendritic Cells, and Tregs. The top 15 upregulated and downregulated genes are signified by red dots. Fig. 6. Comparison of gene expression in immune cells between irradiated and unirradiated Tumors. (A) Heatmap showing gene expression differences in irradiated versus unirradiated tumors treated with NBTXR3+XRT+αPD1. (B) Heatmap indicating differential gene expression in irradiated versus unirradiated tumors subjected to NBTXR3+PRT+αPD1. (C) Differential gene expression in immune cells from irradiated versus unirradiated tumors treated with NBTXR3+XRT+αPD1. (D) Differential gene expression in immune cells in irradiated versus [file 12951_2024_2855_MOESM1_ESM.docx]

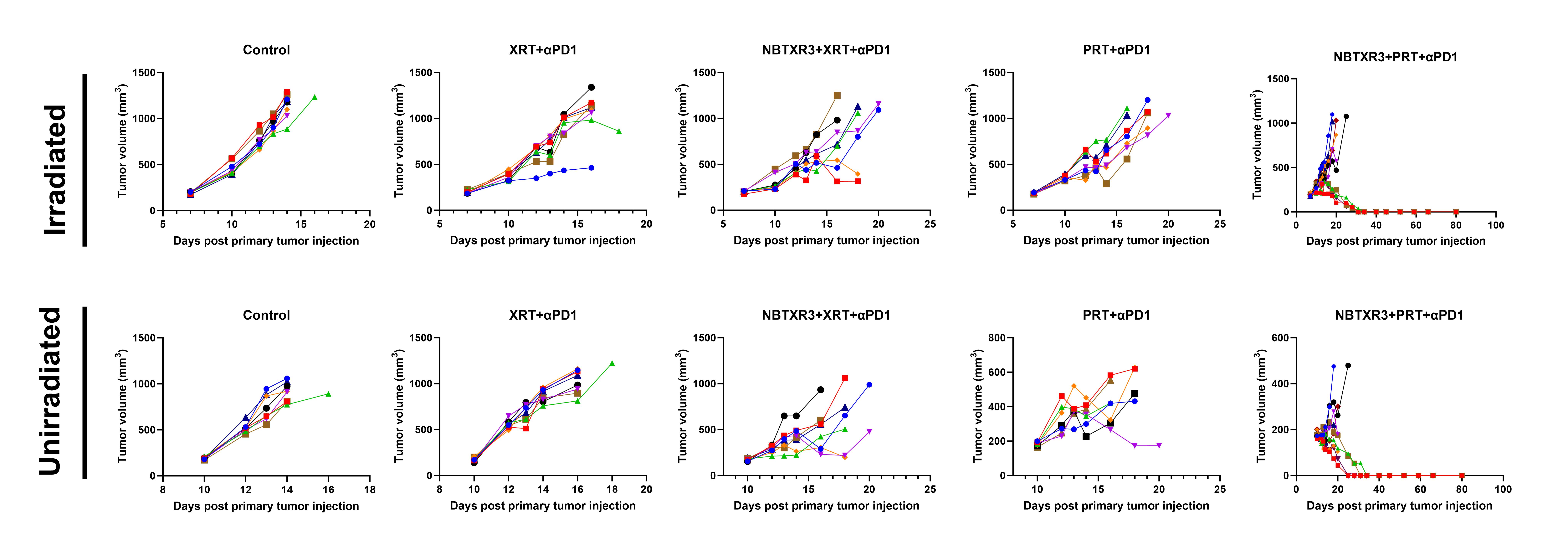


**Supplemental Fig. 1. Individual tumor growth in irradiated and unirradiated tumors subjected to diverse combination therapies involving NBTXR3, XRT, PRT, and αPD1.**

**
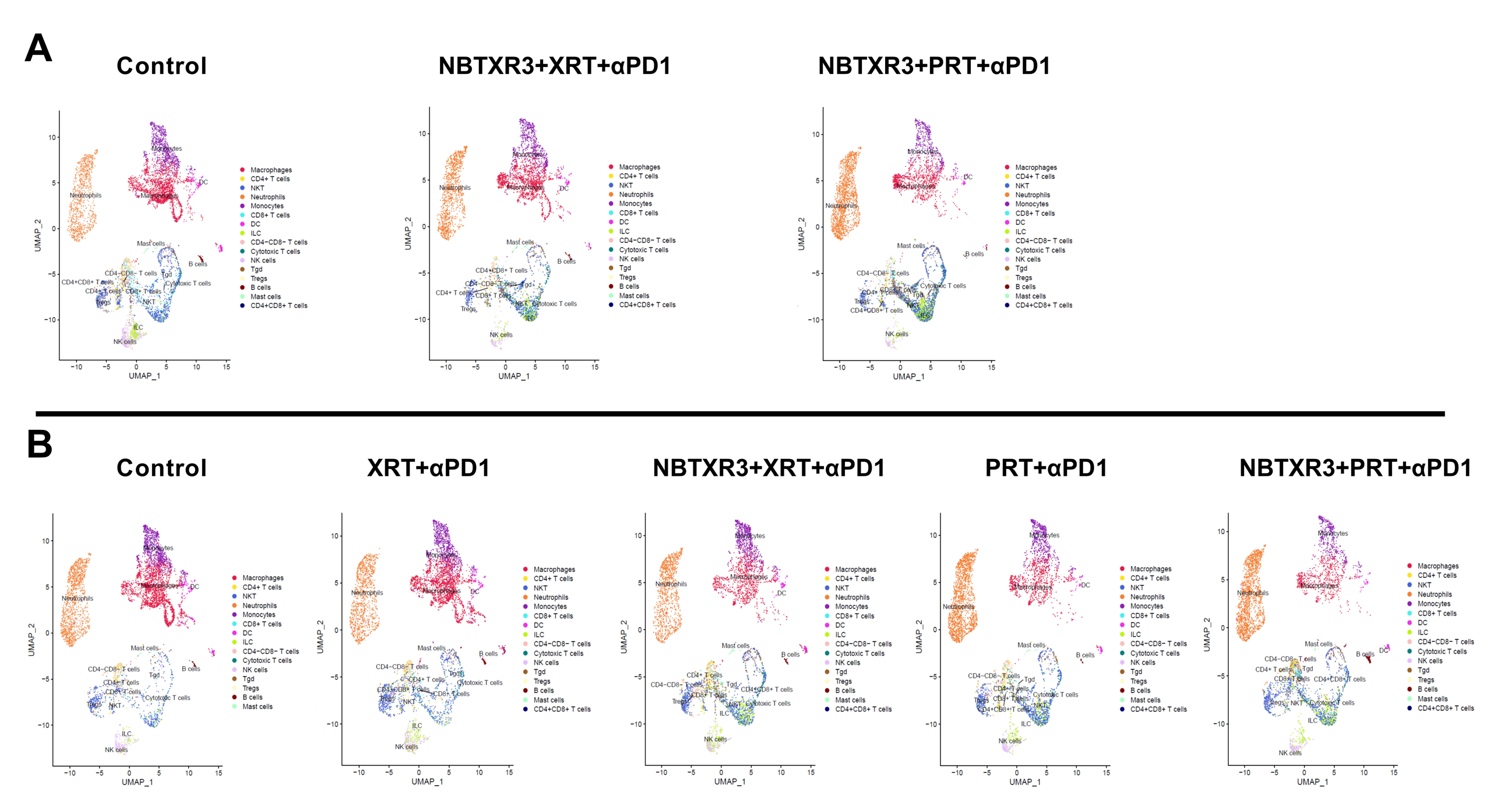
**

**Supplemental Fig. 2. UMPAs of immune cell populations in tumors subjected to different combination therapies of NBTXR3, XRT, PRT, and αPD1.** **(A)** Irradiated tumors. **(B)** Unirradiated tumors.


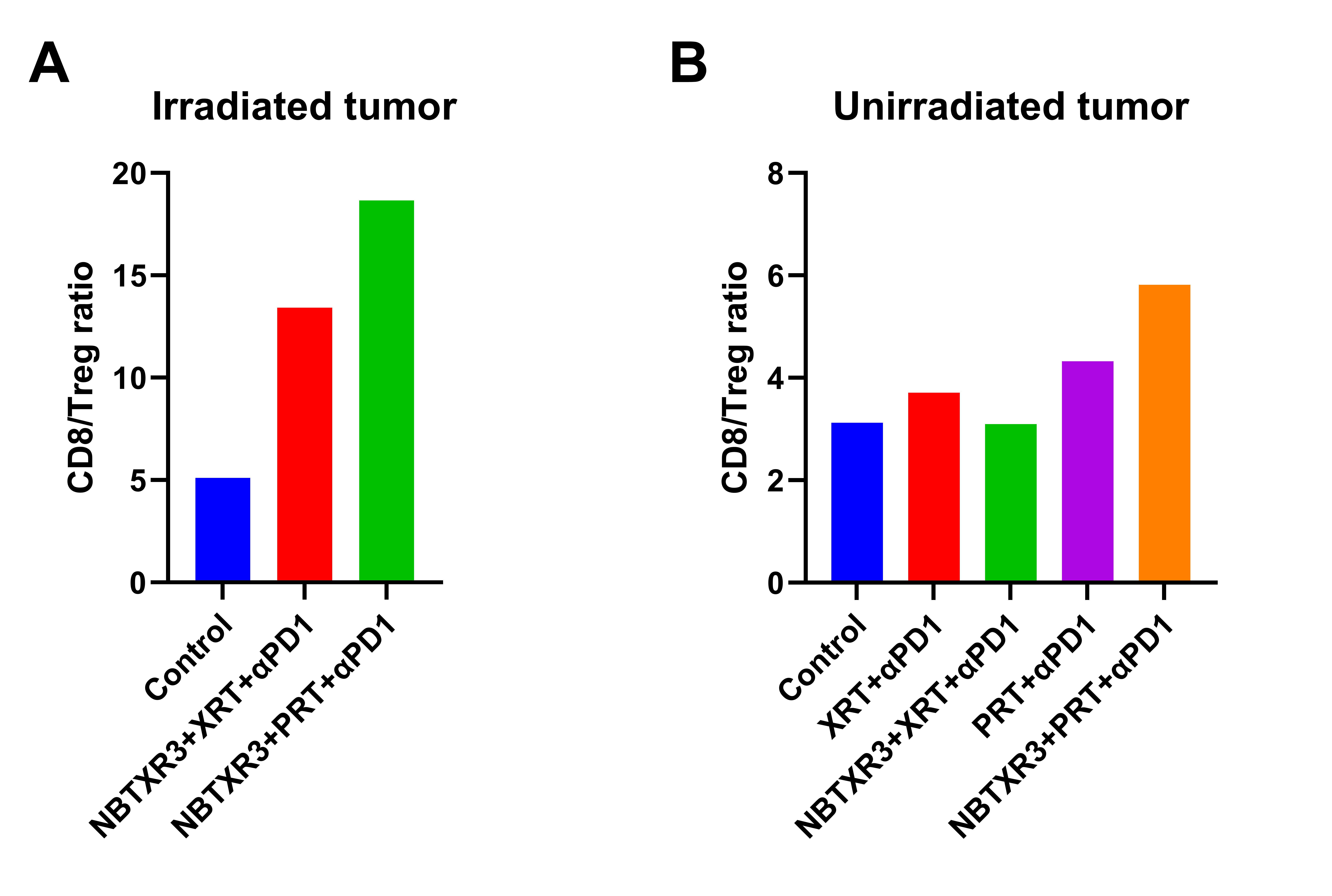


**Supplemental Fig. 3. CD8/Treg ratio.** **(A)** Irradiated tumors. **(B)** Unirradiated tumors.

**

**

**Supplemental Fig. 4. Heatmap illustrating variations in gene expression across diverse immune cells. (A)** Differentiated gene expression in irradiated tumors treated with the NBTXR3+PRT+αPD1 combination to those treated with the NBTXR3+XRT+αPD1 combination. **(B)** Differentiated gene expression in unirradiated tumors subjected to NBTXR3+PRT+αPD1 versus NBTXR3+XRT+αPD1 treatments. **(C)** Differentiated gene expression in unirradiated tumors treated with PRT+αPD1 versus XRT+αPD1.





**Supplemental Fig. 5. Differential gene expression in immune cells across various IRTs. (A)** Irradiated tumors under NBTXR3+XRT+αPD1 versus control. **(B)** Irradiated tumors under NBTXR3+PRT+αPD1 versus control. **(C)** Unirradiated tumors under XRT+αPD1 versus control. **(D)** Unirradiated tumors under NBTXR3+XRT+αPD1 versus control. **(E)** Unirradiated tumors under PRT+αPD1 versus control. **(F)** Unirradiated tumors under NBTXR3+PRT+αPD1 versus control. Each panel focuses on Cytotoxic T Cells, NKT Cells, Dendritic Cells, and Tregs. The top 15 upregulated and downregulated genes are signified by red dots





**Supplemental Fig. 6. Comparison of gene expression in immune cells between irradiated and unirradiated Tumors.** **(A)** Heatmap showing gene expression differences in irradiated versus unirradiated tumors treated with NBTXR3+XRT+ αPD1. **(B)** Heatmap indicating differential gene expression in irradiated versus unirradiated tumors subjected to NBTXR3+PRT+ αPD1. **(C)** Differential gene expression in immune cells from irradiated versus unirradiated tumors treated with NBTXR3+XRT+ αPD1. **(D)** Differential gene expression in immune cells in irradiated versus unirradiated tumors treated with NBTXR3+PRT+ αPD1. The top 15 upregulated and downregulated genes are signified by red dots


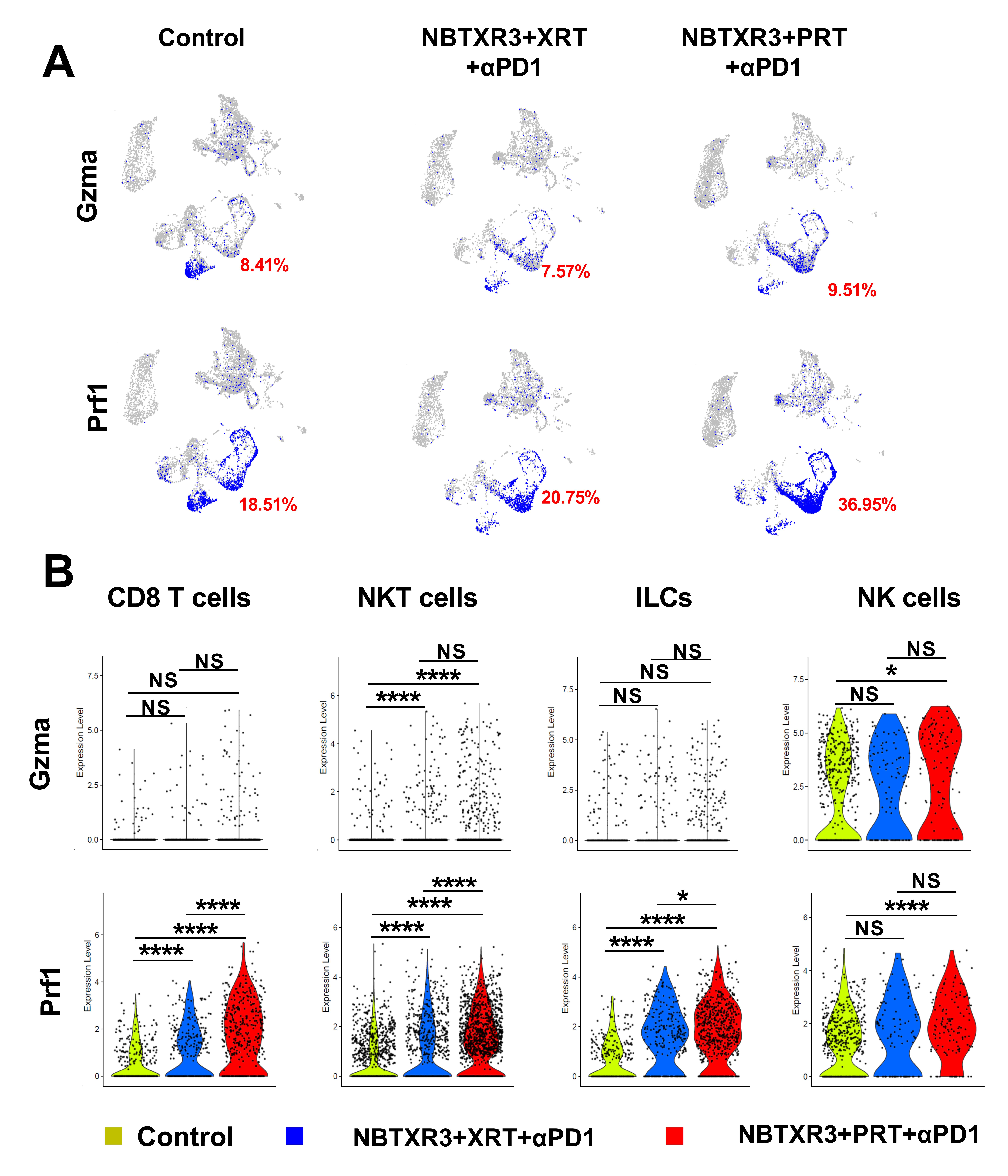


**Supplemental Fig. 7. Gzma and Prf1 expression in irradiated tumors.** **(A)** UMAP of Gzma and Prf1 expression within irradiated tumors. **(B)** Gzma and Prf1 expression levels in irradiated tumors across the following groups: Control, NBTXR3+XRT+ αPD1, and NBTXR3+PRT+ αPD1.





**Supplemental Fig. 8. Gzma and Prf1 expression in unirradiated tumors.** **(A)** UMAP of Gzma and Prf1 expression within unirradiated tumors. **(B)** Gzma and Prf1 expression levels in unirradiated tumors across the following groups: Control, XRT+ αPD1, NBTXR3+XRT+ αPD1, PRT+ αPD1, and NBTXR3+PRT+ αPD1.





**Supplemental Fig. 9. Analysis of checkpoint receptor expression in CD4 and CD8 T Cells.** **(A)** Irradiated tumors. **(B)** Unirradiated tumors.





**Supplemental Fig. 10. UMAP visualization of Tgfb1 and Tgfbi expression.** **(A)** Irradiated tumors. **(B)** Unirradiated tumors.





**Supplemental Fig. 11. Analysis of TNFα expression.** **(A)** Irradiated tumors. **(B)** Unirradiated tumors.


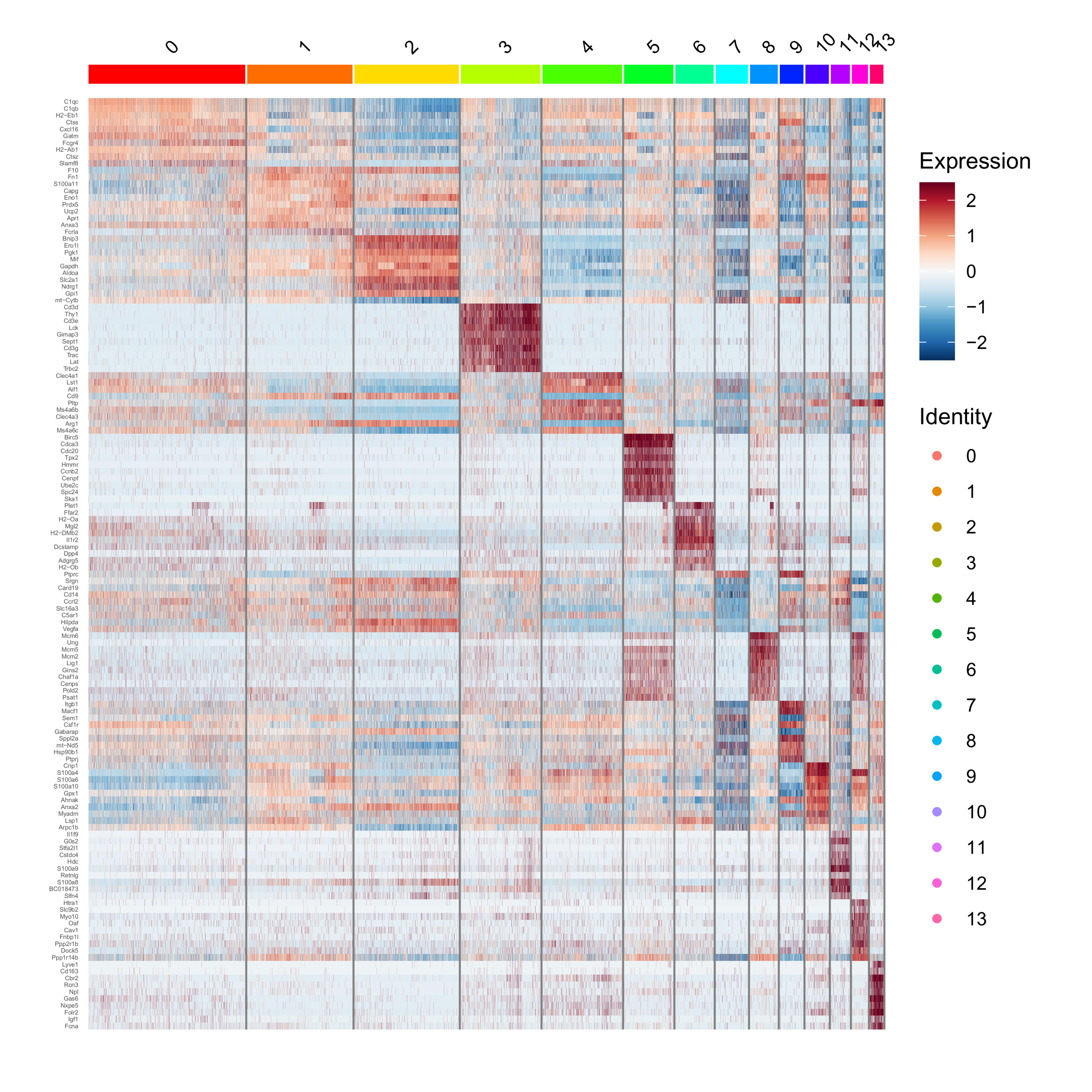


**Supplemental Fig. 12. Heatmap of macrophage clustering.**

**

**

**Supplemental Fig. 13. UMAPs of macrophage subclusters across different treatments.** **(A)** Irradiated tumors. **(B)** Unirradiated tumors.


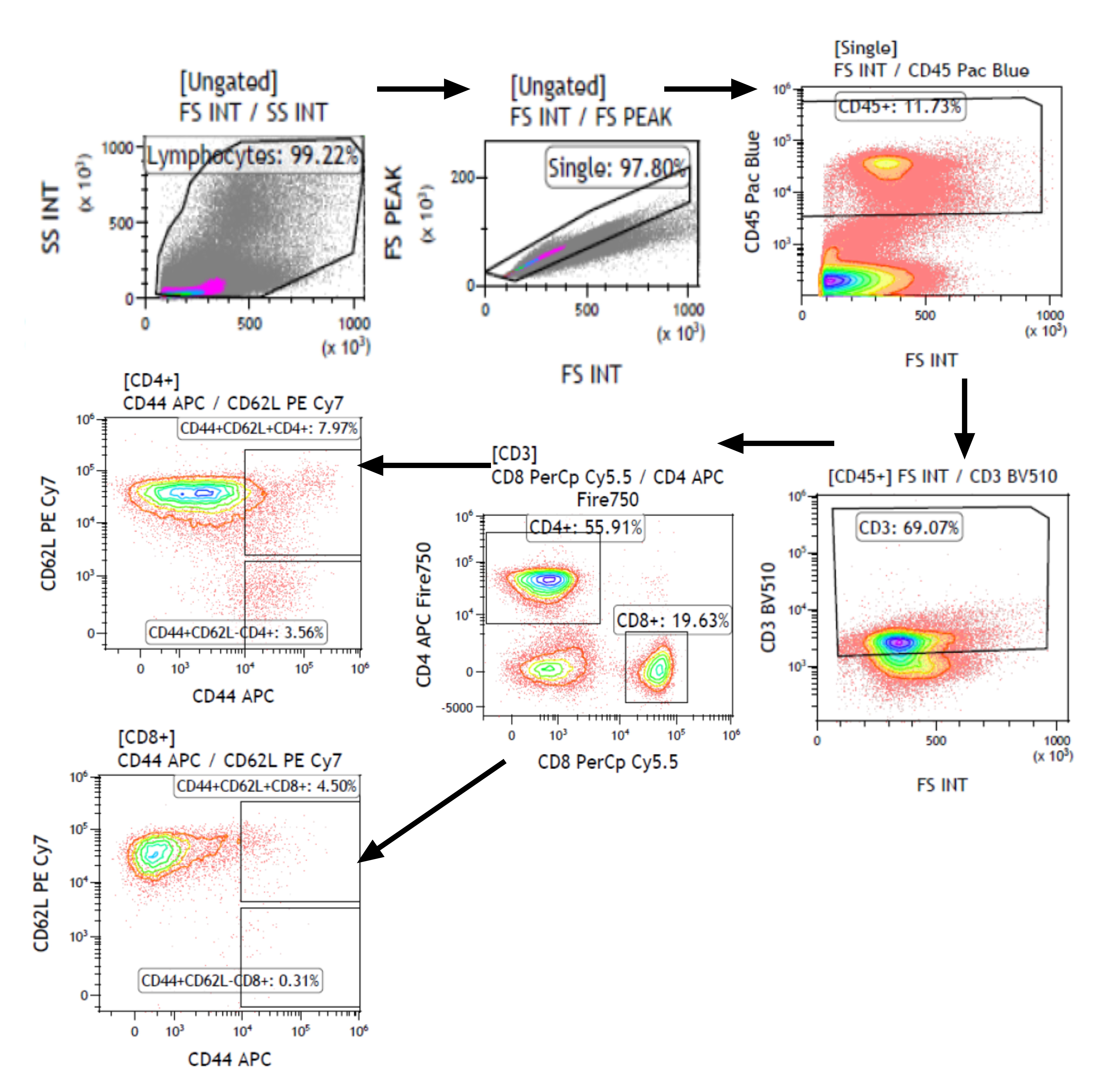


**Supplemental Fig. 14. Flow cytometry gating strategy for identifying memory T cells.**
